# Effect of Vitamin C on bleeding and pain after kidney biopsy: a randomized controlled trial

**DOI:** 10.1186/s12916-026-05037-x

**Published:** 2026-07-03

**Authors:** Jipeng Li, Fang Zhao, Rui Lu, Yang Zha, Fengpei Chang, Xiayin Li, Jingli Gao, Xi Shen, Qing Jia, Jing Zhao, Xiaolan Che, Xiaomeng Xu, Mengting Wu, Hongyi Zhang, Yuting Zhang, Ye Chen, Xinrui Tan, Lijie He

**Affiliations:** 1 Department of Nephrology, Xijing Hospital, Fourth Military Medical University, Xi’an, Shaanxi China; 2 The Graduate School, Xi’an Medical University, Xi’an, Shaanxi China

**Keywords:** Vitamin C, Percutaneous renal biopsy, Randomized controlled trial, Hematoma, Post-procedural pain

## Abstract

**Background:**

Percutaneous renal biopsy (PRB) is an essential diagnostic procedure for kidney diseases, yet postbiopsy perirenal hematoma and pain remain frequent complications. Vitamin C has emerging evidence supporting its role in reducing perioperative bleeding and pain, but its efficacy in the context of PRB is unknown.

**Methods:**

This single-center, prospective, open-label, randomized controlled trial enrolled 710 adults with chronic kidney disease (CKD) scheduled for an ultrasound-guided native kidney biopsy. Participants were randomly assigned (1:1) to receive perioperative intravenous vitamin C (20 mg/kg at 6 h before and 18 h after biopsy) or standard care alone. The primary outcomes were the area of perirenal hematoma on renal ultrasound at 24 h and self-reported pain intensity (Visual Analogue Scale, VAS) at 2, 12, and 24 h post-biopsy.

**Results:**

All 710 randomized participants were included in the analysis. The adjusted mean difference in hematoma area at 24 h was 4.44 mm² (95% CI, −52.31 to 61.18; P = 0.878). After multiple testing correction, there were no significant differences in the primary pain outcome (VAS scores) between groups at any time point; however, an exploratory analysis of pain severity categories indicated a less favorable distribution in the Vitamin C group at 12 h (P for trend =0.033). The incidence of any perirenal hematoma was 56.3% in the vitamin C group versus 50.1% in the control group (risk ratio, 1.12; 95% CI, 0.98–1.29; P = 0.098). Rates of other bleeding complications and adverse events were low and comparable between groups.

**Conclusions:**

In CKD patients undergoing PRB, perioperative intravenous vitamin C did not reduce the size of perirenal hematoma, alleviate postbiopsy pain, or decrease the incidence of bleeding complications within 24 h. These findings do not support the routine use of vitamin C for preventing complications after kidney biopsy.

**Supplementary Information:**

The online version contains supplementary material available at https://doi.org/10.1186/s12916-026-05037-x.

## Background

Percutaneous renal biopsy (PRB) is an indispensable diagnostic procedure for a wide spectrum of kidney diseases. While generally safe under ultrasound guidance, it carries an inherent risk of procedure-related complications, most notably perirenal hematoma and localized pain [1, 2]. Post-biopsy pain primarily stems from direct mechanical injury to the local tissues or irritation caused by perirenal bleeding. Although most perirenal hematomas are small and the associated discomfort is transient and self-limiting, severe hematomas can lead to significant pain, anemia, hemodynamic instability, and in rare instances, necessitate invasive interventions such as transfusion or angioembolization [3, 4]. Despite advances in technique and imaging, there remains a need for safe, effective, and accessible pharmacological strategies to mitigate these risks, with a particular focus on preventing significant bleeding, which is a fundamental contributor to post-procedural morbidity.

The search for an optimal prophylactic agent has yielded limited and inconsistent evidence. Although desmopressin may reduce postbiopsy hematoma incidence [5], concerns remain regarding its safety profile, including a lack of clear postoperative bleeding benefit [6], associations with thrombotic events such as infarction [7], and the risk of provoking severe hyponatremia [8]. Conversely, the antifibrinolytic agent tranexamic acid has, in some studies, unexpectedly been associated with increased hematoma size [9], highlighting the complexity of modulating hemostasis in this setting. This gap underscores the necessity for alternative therapeutic pathways that are both effective and inherently safe.

Vitamin C or ascorbic acid has emerged as a promising candidate for mitigating post-biopsy complications, supported by a compelling biological rationale that extends to hemostasis and analgesia. On one hand, it promotes hemostasis by enhancing collagen synthesis in vessel walls [10] and protecting platelet function [11]. On the other hand, it may also help alleviate pain through its potential antioxidant and anti-inflammatory properties [12], which could mitigate tissue irritation and nociceptive signaling at the puncture site. Thus, with its potential to both reduce bleeding and alleviate pain, vitamin C may offer a broader therapeutic benefit compared to agents targeting hemostasis alone.

Previous clinical evidence have provided preliminary support. For instance, intravenous vitamin C has been associated with decreased bleeding events in gynecological and endoscopic surgeries [13, 14]. Similarly, several randomized trials and meta-analyses suggest that perioperative vitamin C supplementation can lower early postoperative pain scores [15, 16]. However, these findings remain heterogeneous and may be influenced by factors such as surgical setting, dosage, and administration timing. More importantly, no study to date has specifically evaluated whether vitamin C can reduce procedure-related bleeding and pain following PRB.

Therefore, we designed a prospective randomized controlled trial to evaluate the efficacy and safety of perioperative intravenous vitamin C in participants undergoing PRB. The primary objectives are to assess its ability to reduce the incidence and severity of bleeding complications and to alleviate biopsy-related pain. This investigation aims to provide a novel, safe, and cost-effective adjuvant strategy to enhance recovery after PRB. The findings may provide high-quality evidence for the clinical feasibility of perioperative vitamin C use in such minimally invasive procedures.

## Methods

### Study conduct

This study was conducted in the Department of Nephrology at a large tertiary teaching hospital in China, and strictly adhered to the principles of the Declaration of Helsinki and relevant laws and regulations in China. The research protocol was approved by the institutional ethics committee and followed its stipulated requirements.

### Design and participants

This was a single-center, prospective, open-label, randomized controlled trial with two parallel groups (allocation ratio 1:1). This study was in full compliance with the principles of the Declaration of Helsinki. The protocol is registered at the Chinese Clinical Trial Registry (ChiCTR2500106962).

The study enrolled adults with chronic kidney disease (CKD) who were scheduled for a percutaneous ultrasound-guided native kidney biopsy at the study site. Key exclusion criteria included a known hypersensitivity to vitamin C, active bleeding disorders, uncontrolled severe hypertension (systolic BP > 180 mmHg), structural kidney abnormalities, pregnancy, lactation, or oliguria. Full inclusion and exclusion criteria are provided in the protocol (Additional file 2).

### Interventions

Eligible participants were randomized to either the intervention or control group. The intervention group received intravenous vitamin C (20 mg/kg, diluted in 100 mL of 0.9% sodium chloride) administered twice: at 6 h before and 18 h after the biopsy. The control group received standard care without the vitamin C infusion. The vitamin C dose was selected based on pharmacokinetic studies [17] and previous perioperative trials [13, 14].

Under ultrasound guidance, renal lower pole biopsy was conducted in the lateral decubitus position with local anesthesia using 5 mL of 2% lidocaine and a 16 G Tru‑Cut needle. After sterile gauze dressing and abdominal binder compression, patients remained supine for 8 h postoperatively. All procedures were performed by a single experienced senior specialist and her team to ensure operational standardization, based on practice guidelines [18] and local post-procedure protocol.

### Outcomes

Primary Outcomes included: 1) The area of perirenal hematoma assessed by renal ultrasound at 24 h post-biopsy. 2) Pain intensity measured using a self-reported 100 mm visual analogue scale (VAS) at 2, 12, and 24 h.

Secondary Outcomes included the incidence and severity distribution of perirenal hematoma, the incidence of other bleeding events (gross hematuria, transfusion requirement or intervention for bleeding), the distribution of pain severity categories, and the incidence of adverse events (AEs).

### Sample size

The sample size was calculated based on preliminary data from a previous trial (See Protocol). To detect a difference in hematoma area with 80% power at a two-sided alpha of 0.05, and accounting for a potential 20% reduction in effect size, a total of 710 participants (355 per group) were required.

### Randomization and allocation

A computer-generated random sequence was created by an independent statistician using SPSS software. Participants were assigned a sequential study number upon enrollment, which corresponded to their pre-determined group allocation.

### Blinding

This trial was conducted open-label. Participants and treating clinicians were aware of the treatment assignment. However, the primary outcome assessors (radiologists measuring hematoma area on ultrasound) were blinded to group allocation.

### Statistical methods

Analyses followed the intention-to-treat (ITT) principle. Continuous variables were compared using t-tests or Mann-Whitney U tests. Categorical variables were compared using chi-square or Fisher’s exact tests. The Cochran-Mantel-Haenszel (CMH) test was used to analyze trends in ordered categorical outcomes. For the co-primary pain outcome, VAS scores were analyzed both as continuous variables with Bonferroni correction for multiple time points and as ordinal categories. An analysis of covariance (ANCOVA) and a zero-inflated negative binomial regression model was pre-specified as a sensitivity analysis for hematoma area and VAS scores to adjust the baseline confounder of age, respectively. A post‑hoc exploratory subgroup analysis was performed based on baseline urinary vitamin C levels (tertiles and quartiles). The statistical methods are detailed in Supplementary Methods (Additional file 1). A two-sided P-value < 0.05 was considered significant. Analyses were performed using SPSS or R software.

## Results

### Patient enrollment and intervention implementation

Between August and December 2025, a total of 847 participants were consecutively screened for eligibility. Of these, 710 were enrolled and randomly assigned in a 1:1 ratio to either the Vitamin C group (n = 355) or the control group (n = 355). The majority of exclusions during screening were due to failure to meet the inclusion criteria.

All participants received intramuscular administration of 2 units of hemocoagulase bothrops atrox before the biopsy. Participants allocated to the Vitamin C group were scheduled to receive two intravenous infusions of vitamin C (20 mg/kg each): the first dose 6 h before the biopsy and the second dose 18 h after the biopsy. Participants in the control group received no additional prophylactic hemostatic agents and no cross-over between groups occurred.

**Fig. 1:**
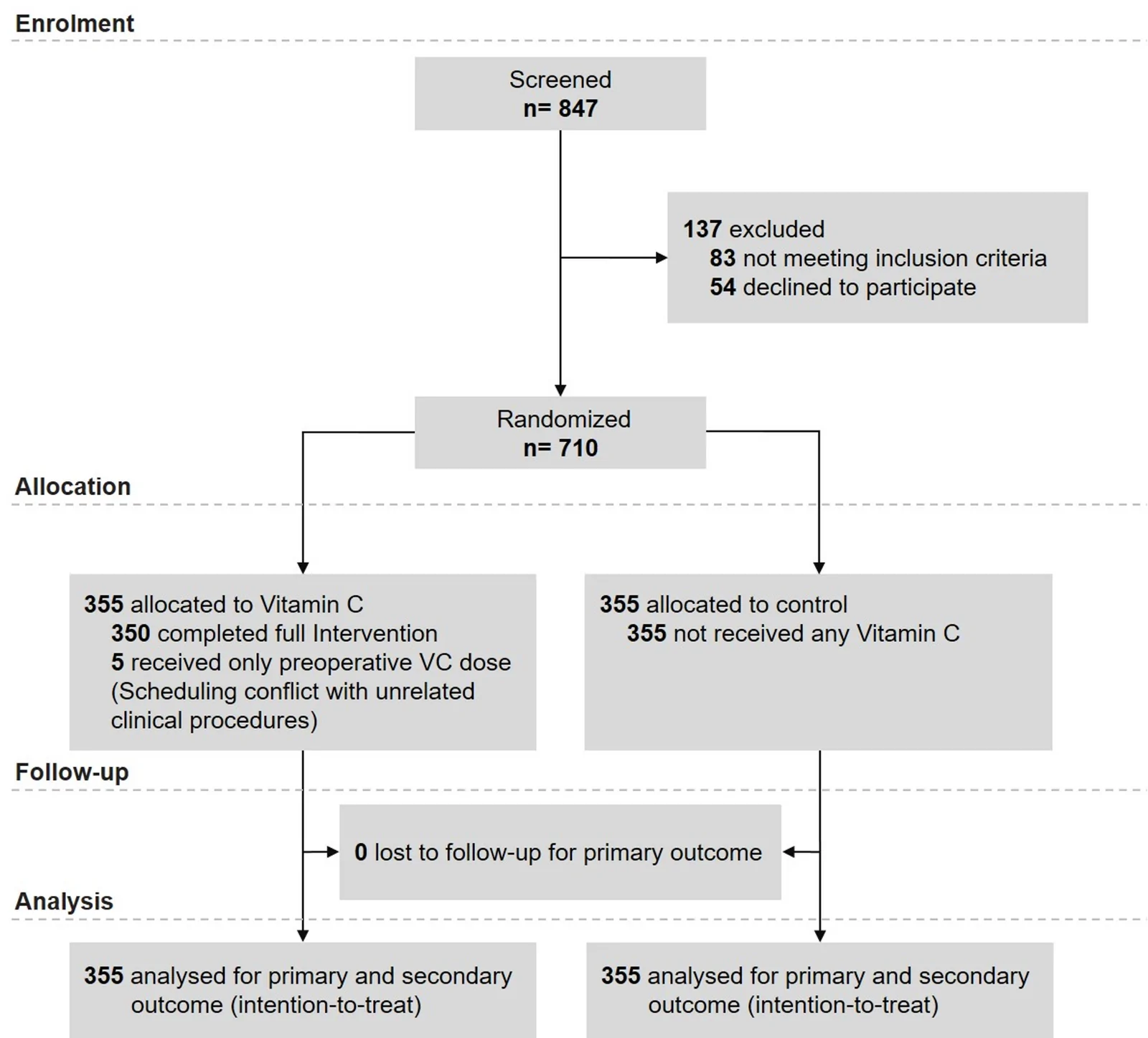
Study patient disposition

A protocol deviation occurred in five participants (1.41%) in the Vitamin C group, who did not receive the second postoperative dose due to missing the predefined administration window after leaving the study unit for required diagnostic tests. All patients completed postoperative renal ultrasound examinations and the Pain VAS self-assessment as scheduled. No participants were lost to follow-up for the primary and secondary outcomes. All 710 participants were included in the ITT analysis. A summary of patient disposition is presented in Fig. 1.

### Demographics and baseline characteristics

Baseline characteristics were presented in Table 1 and Additional file 1: Table S1. The mean age was 46.15 ± 14.67 years in the Vitamin C group and 49.25 ± 14.25 years in the control group (P = 0.004). All other demographic, laboratory, and clinical parameters, medical histories, and the use of concomitant medications were similarly distributed. Although age differed statistically between groups, the effect sizes were small (standardized mean difference: 0.214), suggesting limited clinical relevance. Overall, baseline characteristics were well-matched, supporting the comparability of the two groups for subsequent efficacy analyses.

**Table 1:** Demographics and baseline characteristics

Characteristic	Vitamin C	Control
	N = 355	N = 355
Age at screening, yr	46.15 ± 14.67	49.25 ± 14.25
Female sex, n (%)	146 (41.1)	145 (40.8)
BMI, kg/m^2^	24.81 ± 3.99	24.67 ± 3.75
Blood pressure, mm Hg		
Systolic	126.30 ± 14.72	125.92 ± 16.26
Diastolic	79.91 ± 9.57	80.75 ± 10.18
Albumin, g/dl	3.91 [3.30, 4.40]	3.89 [3.06, 4.44]
Urea, mg/dl	33.24 [24.76, 49.28]	35.58 [27.35, 52.41]
Creatinine, mg/dl	0.92 [0.69, 1.50]	0.93 [0.71, 1.52]
Uric acid, mg/dl	6.05 ± 1.93	6.14 ± 1.96
Cystatin C, mg/dl	0.12 [0.09, 0.17]	0.12 [0.09, 0.17]
eGFR, mL/min per 1.73m^2^	94.18 [51.83, 116.05]	87.37 [47.72, 109.23]
Cholesterol, mg/dl	182.52 [147.72, 227.77]	189.87 [153.91, 237.43]
Triglycerides, mg/dl	165.63 [110.71, 224.45]	150.57 [111.60, 224.08]
Glucose, mg/dl	52.3 [47.7, 59.8]	52.8 [47.6, 58.2]
24-hour urine protein, g	1.49 [0.76, 3.37]	1.71 [0.78, 4.21]
UACR, g/g	1.02 [0.39, 2.81]	1.37 [0.41, 3.64]
PT, s	8.76 ± 0.58	8.77 ± 0.62
APTT, s	31.32 ± 3.79	31.80 ± 4.19
TT, s	18.11 ± 1.22	18.11 ± 1.30
INR	0.97 ± 0.06	0.97 ± 0.07
Medical History, n (%)		
Hypertension	160 (45.1)	182 (51.3)
Diabetes mellitus	66 (18.6)	66 (18.6)
CVD	33 (9.3)	32 (9.0)
Concomitant Medications, n (%)		
Antihypertensive agents	271 (76.3)	280 (78.9)
Glucose-lowering agents	113 (31.8)	94 (26.5)
Lipid-modifying agents	156 (43.9)	146 (41.1)
Immunosuppressants	38 (10.7)	42 (11.8)
Anemia & Bone Mineral Metabolism	71 (20.0)	72 (20.3)

Continuous data are presented as mean ± SD (normally distributed) or median [IQR] (non-normally distributed). Categorical data are presented as n (%). P values for continuous variables are from t-tests or Mann-Whitney U tests, and from χ² or Fisher’s exact tests for categorical variables

*BMI*, body mass index; *UACR*, urine albumin-to-creatinine ratio; *PT*, prothrombin time; *APTT*, activated partial thromboplastin time; *TT*, thrombin time; *INR*, international normalized ratio; *CVD*, cardiovascular disease

### Biopsy procedure and pathological diagnoses

Biopsy procedural details and the spectrum of pathological diagnoses were balanced between the two groups (Table 2 and Additional file 1: Table S2). The majority of biopsies required two needle insertions (Vitamin C: 98.6%; Control: 97.2%), and all but two procedures (both in the control group) were performed on the right kidney. The most common pathological diagnosis in both groups was IgA nephropathy (31.3% each), followed by membranous nephropathy (Vitamin C: 19.4%; Control: 24.2%). The distribution of all other diagnostic categories was similar between groups (all P > 0.05).

**Table 2:** Biopsy information and pathological diagnoses

Characteristic, n (%)	Vitamin C	Control	P value
	N = 355	N = 355	
**Needle insertions**			
2	350 (98.6)	345 (97.2)	0.192
3	5 (1.4)	10 (2.8)	
**Left renal biopsy**	0	2 (0.6)	0.499
**Primary Renal Diagnosis** ^a^			
IgA nephropathy	111 (31.3)	103 (31.3)	0.513
Membranous nephropathy	69 (19.4)	86 (24.2)	0.122
Diabetic nephropathy	47 (13.2)	38 (10.7)	0.298
Hypertensive renal disease^b^	34 (9.6)	21 (5.9)	0.068
Minimal change disease	15 (4.2)	17 (4.8)	0.718
Focal segmental glomerulosclerosis	12 (3.4)	15 (4.2)	0.556
Lupus nephritis	6 (1.7)	8 (2.3)	0.589
Other glomerulonephritides^c^	47 (13.2)	51 (14.4)	0.663

^a^In cases of multiple diagnostic entries, only the highest-priority diagnosis was selected for classification

^b^Hypertensive renal disease was classified as such only when it was the primary or sole diagnosis

^c^Includes all remaining diagnostic categories with individually low frequencies. Full distribution provided in Supplementary Table 2

### Outcomes

Data for the primary and secondary outcomes were available for all 710 randomized participants.

### Primary outcome

#### Perirenal hematoma area

In Table 3, the unadjusted mean difference in hematoma area at 24 h was 1.65 mm^2^ (95% CI, −54.764 to 58.054; P = 0.954). After adjustment for baseline age imbalance using ANCOVA, the adjusted mean difference was 4.44 mm² (95% CI, −52.31 to 61.18 mm^2^; P = 0.878). The model explained minimal variance (R^2^ < 0.001).

**Table 3:** Primary Outcomes

Analysis model	Vitamin C group	Control group	Effect size (95% CI)	P value
	N = 355	N = 355		
**Perirenal hematoma**				
All Participants				
Any hematoma, n (%)	200 (56.3)	178 (50.1)	RR:1.124 (0.978, 1.290)	0.098
Crude area, mm^2^	191.02 ± 274.89	189.37 ± 466.36	MD:1.645 (−54.764, 58.054)	0.954
Adjusted mean area^a^, mm^2^	192.42	187.97	MD:4.435 (−52.305, 61.176)	0.878
Participants with Hematoma				
Area, mm²	339.06 ± 289.78	377.68 ± 602.87	MD:−38.625 (−132.690, 55.440)	0.420
**Pain VAS scores**				
2 h^b^	0 [0, 9]	0 [0, 6]	–	0.105
12 h^b^	0 [0, 7]	0 [0, 5]	–	0.192
24 h^b^	0 [0, 4]	0 [0, 3]	–	0.534

^a^Adjusted for baseline age by ANCOVA. Values presented are estimated marginal means. Effect size is mean difference (MD) or risk ratio (RR)

^b^Data are presented as median [IQR] and were compared using the Mann-Whitney U test. Adjusted P-values were calculated using the Bonferroni correction for comparisons across 3 time points

#### Pain VAS scores

At all time points, the median VAS score in both groups was 0. The raw P value suggested a difference at 2 h post-biopsy (P = 0.035), whereas the differences at 12 and 24 h were not significant (P = 0.064 and P = 0.178, respectively)(Additional file 1: Table S3). After Bonferroni correction for three tests, none of the comparisons remained statistically significant (adjusted P = 0.105, 0.192, and 0.534, respectively) (Table 3).

A zero-inflated negative binomial regression was performed to account for the substantial proportion of zero scores and to adjust for baseline age (Additional file 1: Table S4 and Figure S4). However, age was a significant influencing factor. Older age was not only associated with a lower expected pain intensity (at 12 h: rate ratio [RR] = 0.984, P = 0.001) but also with reduced odds of reporting zero pain (at 2 h: odds ratio [OR] = 1.019, P < 0.001).

### Secondary outcomes

#### Bleeding events and complications

There were no significant differences between groups in the incidence of perirenal hematoma or other bleeding complications (Table 4). The proportion of participants with any perirenal hematoma was 56.3% in the vitamin C group and 50.1% in the control group (RR, 1.124; 95% CI, 0.978–1.290; P = 0.098). There was no statistically significant difference in the distribution of perirenal hematoma severity between the Vitamin C and Control groups (χ² = 1.542, P = 0.214). The rates of gross hematuria and transfusion requirement were also comparable between groups. No patient in either group required Surgical or interventional surgery for bleeding.

**Table 4:** Secondary outcomes

Outcome, n (%)	Vitamin C	Control	Risk Ratio (95% CI)	P value^a^
	N = 355	N = 355		
**Perirenal hematoma severity**				
Mild (≤200 mm²)	71 (20.0)	61 (17.2)	1.164 (0.855, 1.585)	0.335
Moderate (201–400 mm²)	76 (21.4)	67 (18.9)	1.134 (0.846, 1.522)	0.400
Severe (>400 mm²)	53 (14.9)	50 (14.1)	1.060 (0.742, 1.515)	0.749
P for trend^b^			χ² = 1.542	0.214
**Gross hematuria**	7 (2.0)	2 (0.6)	3.500 (0.732, 16.732)	0.093
**Transfusion required**	0	2 (0.6)	–	0.499
**Intervention for bleeding**	0	0	–	–
**Pain Severity over time**				
**2 h**				
Pain-free (0–4)	238 (67.0)	260 (73.2)	0.915 (0.831, 1.008)	0.071
Mild (5–44)	98 (27.6)	81 (22.8)	1.210 (0.938, 1.561)	0.142
Moderate (45–74)	15 (4.2)	10 (2.8)	1.500 (0.683, 3.294)	0.309
Severe (>75)	4 (1.1)	4 (1.1)	–	–
P for trend^b^			χ² = 2.791	0.095
**12 h**				
Pain-free (0–4)	239 (67.3)	265 (74.6)	0.902 (0.821, 0.991)	0.032
Mild (5–44)	95 (26.8)	77 (21.7)	1.234 (0.949, 1.603)	0.115
Moderate (45–74)	18 (5.1)	10 (2.8)	1.800 (0.843, 3.845)	0.123
Severe (>75)	3 (0.8)	3 (0.8)	–	–
P for trend^b^			χ² = 4.567	0.033
**24 h**				
Pain-free (0–4)	277 (78.0)	285 (80.3)	0.972 (0.901, 1.048)	0.460
Mild (5–44)	72 (20.3)	66 (18.6)	1.091 (0.808, 1.472)	0.569
Moderate (45–74)	6 (1.7)	3 (0.8)	2.000 (0.504, 7.935)	0.505
Severe (>75)	0	1 (0.3)	–	–
P for trend^b^			χ² = 0.549	0.459

^a^P value for between-group comparison, χ² or Fisher’s exact test

^b^Cochran-Mantel-Haenszel test for linear trend (χ² = 1.542, df = 1)

#### Postbiopsy pain severity trends

The distribution of local pain severity at 2, 12, and 24 h postoperatively is detailed in Table 4. There was a statistically significant difference in the pain severity distribution at 12 h (P for trend = 0.033), favoring the control group which had a higher proportion of pain-free participants (74.6% vs. 67.3%). At 2 h, a non-significant trend was observed (P = 0.095). By 24 h, the distribution between groups was similar, with no evidence of a linear trend (P = 0.459).

#### Changes in laboratory and vital sign parameters

Postbiopsy urinary vitamin C concentration increased significantly in the vitamin C group compared with the control group (Diff, 25.67 ± 18.09 mg/L vs. −1.01 ± 9.66 mg/L; SMD, 1.839; 95% CI, 1.663 to 2.014; P < 0.001), confirming adherence to the protocol. No significant between-group differences were observed in the postbiopsy changes from baseline in systolic blood pressure, hemoglobin level, or platelet count (Table 5).

**Table 5:** Changes in post-laboratory and vital sign parameters

Parameter	Time Point	Vitamin C	Control	Between-Group Difference	P value
		N = 355	N = 355	SMD (95% CI)	
Urinary Vitamin C, mg/l	Baseline	32.10 ± 13.11	32.68 ± 16.15	−0.040 (−0.187, 0.107)	0.596
	Postbiopsy	57.76 ± 25.00	31.67 ± 16.67	1.228 (1.067, 1.388)	<0.001
	Diff.	25.67 ± 18.09	−1.01 ± 9.66	1.839 (1.663, 2.014)	<0.001
Systolic BP, mm Hg	Baseline	126.30 ± 14.72	125.92 ± 16.26	0.024 (−0.123, 0.171)	0.749
	Postbiopsy	123.48 ± 16.26	124.48 ± 17.23	−0.060(−0.207,0.087)	0.427
	Diff.	−2.81 ± 12.80	−1.44 ± 14.72	−0.100 (−0.247, 0.048)	0.185
Hemoglobin, g/dl	Baseline	13.50 ± 2.47	13.36 ± 2.52	0.054 (−0.093, 0.201)	0.469
	Postbiopsy	13.16 ± 2.27	12.96 ± 2.41	0.086 (−0.061, 0.233)	0.251
	Diff.	−0.34 ± 1.10	−0.40 ± 1.07	0.061 (−0.087, 0.208)	0.419
Platelet, 10^9^/l	Baseline	227.20 ± 71.91	228.11 ± 74.24	−0.012 (−0.160, 0.135)	0.869
	Postbiopsy	222.03 ± 68.37	221.89 ± 72.58	0.002 (−0.145, 0.149)	0.978
	Diff.	−5.17 ± 47.17	−6.22 ± 48.15	0.022 (−0.125, 0.169)	0.769

#### Subgroup analyses

In post‑hoc exploratory subgroup analyses (Additional file 1: Table S7–S9), we observed no significant interaction between treatment and baseline urinary vitamin C levels for the primary outcome of hematoma area (tertiles p = 0.422; quartiles p = 0.484) or for pain outcomes (all p for interaction >0.05). No subgroup benefited from vitamin C.

#### Adverse events

Adverse events were monitored from the first administration of the intervention until the end of study. The overall incidence of AEs was low and comparable between the two groups: 4.2% (15 of 355) in the Vitamin C group and 3.7% (13 of 355) in the control group (Table 6 and Additional file 1: Table S5). Gastrointestinal disorders were the most frequently reported events. All reported AEs were Grade 1 or 2 (Additional file 1: Table S6). No serious AEs were reported, and no specific AEs associated with Vitamin C, such as hemolysis, nephrolithiasis, or allergic reactions, were observed.

**Table 6:** Adverse events during the study period

Preferred Term, n (%)	Vitamin C	Control
	N = 355	N = 355
Any adverse events	15 (4.23)	13 (3.66)
Gastrointestinal Disorders	9 (2.54)	5 (1.41)
General Conditions and Administration Site Conditions	2 (0.56)	2 (0.56)
Respiratory, Thoracic and Mediastinal Disorders	0	1 (0.28)
Musculoskeletal and Connective Tissue Disorders	3 (0.85)	1 (0.28)
Nervous System Disorders	3 (0.85)	3 (0.85)
Renal and Urinary Disorders	0	2 (0.56)
Psychiatric Disorders	0	1 (0.28)

AE grading followed CTCAE v5.0. Incidence (%) = (n/N) × 100, where n is the number of patients with the AE

## Discussion

This was a prospective, open-label, randomized controlled trial designed to evaluate the efficacy and safety of intravenous vitamin C for preventing bleeding and pain complications after PRB in CKD patients. The intervention regimen was based on the pharmacokinetic profile of vitamin C, aiming to maintain effective plasma concentrations. The primary results demonstrated that vitamin C failed to reduce the perirenal hematoma area at 24 h post-biopsy or alleviate pain at puncture site within 24 h.

Our study revealed that, compared with control, vitamin C was not effective in preventing perirenal hematoma after kidney biopsy. The between-group difference was not statistically significant and was clinically negligible. The analysis of pain outcomes presented complexity. No significant difference was found based on the continuous VAS scores after multiple testing correction. However, an exploratory analysis based on ordered categories suggested a trend toward less favorable pain severity distribution in the vitamin C group at 12 h. Regarding safety, all serious adverse events were unrelated to the study intervention, indicating the overall safety of the dosing regimen.

The negative outcomes for both primary endpoints revealed that vitamin C did not provide clinical benefits to CKD patients with PRB in preventing post-biopsy complications. Several factors may explain the gap between these theoretical benefits and our negative findings. While vitamin C can contribute to vascular repair by promoting collagen synthesis [19, 20], or to postoperative oxidative stress and inflammation by inhibiting the NF-κB pathway [21], these are chronic, adaptive processes. In contrast, post-biopsy bleeding and pain are immediate consequences of acute mechanical trauma [22], involving direct vascular injury and a rapid local inflammatory response. Furthermore, the administered dose may have been inadequate to achieve effective tissue concentrations for acute hemostasis or analgesia. The characteristically rapid metabolism and renal clearance of vitamin C could have further limited the efficacy of our short-term perioperative regimen, preventing the maintenance of sufficient concentration during the window following injury. This pharmacokinetic challenge is supported by our observed changes in urinary vitamin C levels before and after the procedure. Thus, while chronic supplementation can improve conditions like gingival bleeding [23], temporary administration of vitamin C during the perioperative period of biopsy was not effective.

Although an exploratory analysis suggested a less favorable distribution of pain severity in the vitamin C group at 12 h, a more robust zero-inflated negative binomial regression model [24], after adjusting for the strong confounder of age, showed no significant association between vitamin C and pain intensity at any time point. This inconsistency may be influenced by changes in pain sensitivity with aging [25]. Generally, our data still do not support a pain-relieving benefit for vitamin C post-biopsy, after effectively eliminating the influence of unbalanced age on pain reporting.

Our study adhered rigorously to a prospective randomized controlled design, providing a high-quality framework. The pharmacokinetic-based intervention regimen enhanced the rigor of the hypothesis testing. Blinded assessment of the primary outcome, with quantitative measurements performed by radiologists unaware of group allocation, significantly minimized measurement bias for the primary endpoint. All randomized participants were included in the ITT analysis, adhering to the study design principles. However, this study has several limitations. The open-label design may have introduced reporting bias. Pain-related analyses across multiple time points were exploratory; although the primary outcome results are robust, findings on pain should be considered hypothesis - generating. Only one dose and schedule of vitamin C was tested. Hematoma area measurement is operator-dependent and pain VAS is subjective, despite our use of blinded assessors. Exclusion of high-risk patients limits generalizability to those with bleeding diatheses, uncontrolled hypertension, or structural kidney abnormalities. Urinary vitamin C concentration does not reliably reflect systemic vitamin C status; although a high urinary level suggests relative sufficiency in some patients, a low level does not necessarily indicate deficiency, and the absence of plasma vitamin C data is a limitation.

Our results contrast with those of earlier, sample-limited interventional studies [13, 14, 16, 26]. This discrepancy may stem from differences in participant populations, the timing and dosing of the intervention or study design. Our findings showed that a short-term, temporary use of vitamin C to prevent bleeding and pain is not recommended for CKD patients undergoing PRB. Given the negative results of this large trial, future research may focus on alternative dosing strategies or higher‑risk populations such as vitamin C‑deficient patients.

## Conclusions

Our study demonstrates that intravenous vitamin C does not prevent bleeding or alleviate pain after kidney biopsy in CKD patients and therefore should not be used routinely in this clinical procedure.

## Supplementary information

Below is the link to the electronic supplementary material.

Supplementary Material 1: dditional File 1: Supplementary Methods, Results, and Definitions. This file contains: Supplementary Methods (post-hoc subgroup analysis),Supplementary Results (Tables S1–S9, Figure S1), and Supplementary Definitions (medical history and biopsy procedure).

Supplementary Material 2: Additional file 2: The full study protocol (v2.0).

## Data Availability

A deidentified participant data that support the findings of this study are available upon request from the corresponding author upon reasonable request, or can access whole data from the Clinical Trial Management Public Platform (ResMan, http://www.medresman.org.cn) within 6 months after posting the result.

## Abbreviations

AE: Adverse Event
ANCOVA: Analysis of Covariance
APTT: Activated Partial Thromboplastin Time
BMI: Body Mass Index
BP: Blood Pressure
CI: Confidence Interval
CKD: Chronic Kidney Disease
CMH: Cochran-Mantel-Haenszel
CTCAE: Common Terminology Criteria for Adverse Events
CVD: Cardiovascular Disease
eGFR: estimated Glomerular Filtration Rate
INR: International Normalized Ratio
ITT: Intention-to-Treat
MD: Mean Difference
OR: Odds Ratio
PRB: Percutaneous Renal Biopsy
PT: Prothrombin Time
RR: Risk Ratio
SMD: Standardized Mean Difference
TT: Thrombin Time
UACR: Urine Albumin-to-Creatinine Ratio
VAS: Visual Analogue Scale
