# Supplementary material for: Effect of Vitamin C on bleeding and pain after kidney biopsy: a randomized controlled trial
Source: BMC Med. 2026 Jul 3;24:515. doi: 10.1186/s12916-026-05037-x (PMC13613656; doi:10.1186/s12916-026-05037-x)
Supplement: Supplementary file 1 [file 12916_2026_5037_MOESM1_ESM.docx]

**Contents**

[1. Supplementary Methods	1](#_Toc17555)

[Post-hoc subgroup analysis based on baseline urinary vitamin C levels	1](#_Toc260)

[2. Supplementary Results	3](#_Toc30145)

[2.1 Baseline and primary analyses	3](#_Toc27535)

[Table S1. Demographics and baseline characteristics of study population	3](#_Toc22410)

[Table S2. Biopsy information and pathological diagnoses	6](#_Toc3864)

[Table S3. Pain VAS Scores Over Time	7](#_Toc2781)

[Table S4. Results of Zero-Inflated Negative Binomial Regression for Pain Intensity	8](#_Toc12658)

[Table S5. Adverse events during the study period (CTCAE v5.0)	9](#_Toc11382)

[Table S6. Summary of Adverse Event Severity graded by CTCAE 5.0	10](#_Toc31186)

[Figure S1. Association of Vitamin C treatment and participants age with postbiopsy pain intensity.	11](#_Toc30327)

[2.2 Post-hoc subgroup analysis	12](#_Toc12937)

[Table S7. Sample sizes for stratification of baseline urinary vitamin C levels	12](#_Toc1983)

[Table S8. Post-hoc subgroup analysis by baseline urinary vitamin C tertiles	12](#_Toc19847)

[Table S9. Post-hoc subgroup analysis by baseline urinary vitamin C quartiles	13](#_Toc18145)

[3. Supplementary Definitions	14](#_Toc27117)

[Medical History Definition	14](#_Toc17899)

[Biopsy Procedure	15](#_Toc19258)

1. Supplementary Methods

# Post-hoc subgroup analysis based on baseline urinary vitamin C levels

**1) Rationale and design**

The following subgroup analyses were not pre-specified in the original study protocol or statistical analysis plan. They were conducted as post-hoc exploratory analyses to examine whether baseline urinary vitamin C status might modify the effect of perioperative intravenous vitamin C on post-biopsy outcomes.

**2) Subgroup definitions**

Baseline urinary vitamin C was used as the stratifying variable. To avoid arbitrary cut-off choices and to assess potential effect modification across the distribution of urinary vitamin C, we created two sets of subgroups:

**- Tertiles:**

Low VC (lowest tertile), Medium VC (middle tertile), High VC (highest tertile). The sample size for each tertile was approximately one‑third of the total cohort (237, 236, and 237 participants, respectively).

**- Quartiles:**

Q1 (lowest), Q2, Q3, Q4 (highest). Each quartile contained approximately 25% of the cohort (178, 177, 177, and 178 participants, respectively).

The cut-off values for tertiles and quartiles were derived from the empirical distribution of baseline urinary vitamin C in the entire study population (N=710). Equal group sizes were achieved by rank‑based stratification.

**3) Outcomes assessed in subgroup analyses**

The same primary and secondary outcomes as in the main analysis were evaluated within each subgroup:

**- Continuous outcomes:** perirenal hematoma area (mm²) at 24 hours, and pain VAS scores at 2, 12, and 24 hours post‑biopsy.

**- Binary outcome:** incidence of any perirenal hematoma (PRH, yes/no).

**4) Statistical methods for subgroup effects**

For each subgroup level, the treatment effect (intervention vs. control) was estimated separately:

**- Continuous outcomes:** Mean difference (intervention minus control) with 95% confidence interval (CI) was calculated using two‑sample t‑tests. The reported effect is the mean of the intervention group minus the mean of the control group, and the CI was transformed accordingly from the t‑test output.

**- Binary outcome:** Odds ratio (OR) for any perirenal hematoma (intervention vs. control) with 95% CI was obtained from univariate logistic regression. The corresponding p‑value was derived from the chi‑square test (or Fisher’s exact test when expected cell counts were <5).

**5) Interaction tests.**

To formally evaluate whether the treatment effect differed across levels of baseline urinary vitamin C, we performed interaction tests for each outcome and each stratification method (tertiles and quartiles).

**- Continuous outcomes:** A linear regression model was fitted with the outcome as the dependent variable, and treatment group, the subgroup variable (tertile or quartile), and their interaction term as independent variables. Type III analysis of variance (ANOVA) was used to obtain the p‑value for the interaction term (group × subgroup).

**- Binary outcome:** Logistic regression models were fitted. The full model included treatment group, the subgroup variable, and their interaction term; the reduced model included only the main effects. A likelihood ratio test compared the two models to derive the p‑value for the interaction.

All models adjusted for the stratification factor itself but not for additional covariates, as the primary aim was to test effect modification by baseline urinary vitamin C without overadjustment. Analyses were performed using R software (version 4.5.2) with the car package for Type III ANOVA. No multiplicity adjustment was applied given the exploratory post‑hoc nature of these analyses.

The results of these post‑hoc subgroup analyses are presented exclusively in the Supplementary Materials (Tables S7–S11). The findings are described briefly in the Results section of the main manuscript with appropriate caveats, and their limitations are discussed in the Discussion section.

2. Supplementary Results

2.1 Baseline and primary analyses

# Table S1. Demographics and baseline characteristics of study population

| **Characteristic** | **Vitamin C** | **Control** | **Effect Size (95% CI)** | **P value** |
| --- | --- | --- | --- | --- |
|  | N=355 | N=355 |  |  |
| **Demographics** |  |  |  |  |
| Age at screening, yr | 46.15±14.67 | 49.25±14.25 | SMD: 0.214 (-0.362, -0.067) | 0.004 |
| Female sex, n (%) | 146 (41.1) | 145 (40.8) | RD%: 0.282 (-6.954, 7.518) | 0.939 |
| BMI, kg/m^2^ | 24.81±3.99 | 24.67±3.75 | SMD: 0.036 (-0.111, 0.183) | 0.631 |
| **Clinical Measurements** |  |  |  |  |
| Blood Pressure, mm Hg |  |  |  | 0.714 |
| Systolic | 126.30±14.72 | 125.92±16.26 | SMD: 0.024 (-0.123, 0.171) | 0.749 |
| Diastolic | 79.91±9.57 | 80.75±10.18 | SMD: -0.085 (-0.232, 0.063) | 0.260 |
| WBC, 10^9^/l | 6.99±2.08 | 6.96±2.28 | SMD: 0.015 (-0.132, 0.162) | 0.845 |
| RBC, 10^12^/l | 4.48±0.80 | 4.47±0.81 | SMD: 0.013 (-0.134, 0.160) | 0.863 |
| HGB, g/dl | 13.50±2.47 | 13.36±2.52 | SMD: 0.054 (-0.093, 0.201) | 0.469 |
| PLT, 10^9^/l | 227.20±71.91 | 228.11±74.24 | SMD: -0.012 (-0.160, 0.135) | 0.869 |
| ALT, IU/l | 19 [13, 30] | 20 [14, 29] | Median Diff: 0 (-1.0, -2.0) | 0.721 |
| AST, IU/l | 19 [16, 25] | 20 [17, 28] | Median Diff: -1.0 (-2.0, 0) | 0.055 |
| Albumin, g/dl | 3.91 [3.30, 4.40] | 3.89 [3.06, 4.44] | Median Diff: 0.050 (-0.070, 0.180 ) | 0.443 |
| Urea, mg/dl | 33.24 [24.76, 49.28] | 35.58 [27.35, 52.41] | Median Diff: -2.162 (-4.444, -0.060) | 0.058 |
| Creatinine, mg/dl | 0.92 [0.69, 1.50] | 0.93 [0.71, 1.52] | Median Diff: -0.023 (-0.090, 0.045) | 0.486 |
| Uric acid, mg/dl | 6.05±1.93 | 6.14±1.96 | SMD: -0.046 (-0.193, 0.102) | 0.544 |
| Cystatin C, mg/dl | 0.12 [0.09, 0.17] | 0.12 [0.09, 0.17] | Median Diff: -0.001 (-0.008, 0.006) | 0.727 |
| eGFR, mL/min per 1.73m^2^ | 94.18 [51.83, 116.05] | 87.37 [47.72, 109.23] | Median Diff: 4.575 (-0.302, 9.298) | 0.064 |
| Cholesterol, mg/dl | 182.52 [147.72, 227.77] | 189.87 [153.91, 237.43] | Median Diff: -7.347 (-15.855, 1.160) | 0.088 |
| Triglycerides, mg/dl | 165.63 [110.71, 224.45] | 150.57 [111.60, 224.08] | Median Diff: 7.971 (-4.429, 20.371) | 0.225 |
| Glucose, mg/dl | 52.3 [47.7, 59.8] | 52.8 [47.6, 58.2] | Median Diff: 0.10 (-1.30, 1.40) | 0.925 |
| 24-hour urine protein, g | 1.49 [0.76, 3.37] | 1.71 [0.78, 4.21] | Median Diff: -0.173 (-0.405, 0.040) | 0.112 |
| UACR, g/g | 1.02 [0.39, 2.81] | 1.37 [0.41, 3.64] | Median Diff: -0.157 (-0.359, 0.012) | 0.070 |
| PT, s | 8.76±0.58 | 8.77±0.62 | SMD: -0.029 (-0.177, 0.118) | 0.695 |
| APTT, s | 31.32±3.79 | 31.80±4.19 | SMD: -0.118 (-0.266, 0.029) | 0.115 |
| TT, s | 18.11±1.22 | 18.11±1.30 | SMD: 0.006 (-0.141, 0.153) | 0.936 |
| INR | 0.97±0.06 | 0.97±0.07 | SMD: -0.008 (-0.155, 0.140) | 0.920 |
| Urinary Vitamin C, mg/l | 32.10±13.11 | 32.68±16.15 | SMD: -0.040 (-0.187, 0.107) | 0.596 |
| **Medical History, n (%)**^*^ |  |  |  |  |
| Hypertension | 160 (45.1) | 182 (51.3) | RD%: -6.198 (-13.267, 0.872) | 0.098 |
| Diabetes Mellitus | 66 (18.6) | 66 (18.6) | - | - |
| CVD | 33 (9.3) | 32 (9.0) | RD%: 0.282 (-3.452, 4.015) | 0.896 |
| Hepatitis B | 8 (2.3) | 9 (2.5) | RD%: -0.282 (-2.423, 1.860) | 0.806 |
| **Concomitant Medications, n (%)** |  |  |  |  |
| Antihypertensive agents | 271 (76.3) | 280 (78.9) | RD%: -2.535 (-8.408, 3.338) | 0.418 |
| Alpha-blockers | 14 (3.9) | 18 (5.1) | RD%: -1.127 (-4.071, 1.816) | 0.469 |
| ACEI/ARB/ARNI | 238 (67.0) | 219 (61.7) | RD%: 5.352 (-1.312, 12.016) | 0.137 |
| Beta-blockers | 49 (13.8) | 42 (11.8) | RD%: 1.972 (-2.947, 6.890) | 0.432 |
| CCB | 114 (32.1) | 127 (35.8) | RD%: -3.662 (-10.304, 2.981) | 0.303 |
| Glucose-lowering agents | 113 (31.8) | 94 (26.5) | RD%: 5.352 (-1.318, 12.023) | 0.117 |
| Insulin | 28 (7.9) | 23 (6.5) | RD%: 1.408 (-2.324, 5.140) | 0.467 |
| Biguanides | 23 (6.5) | 16 (4.5) | RD%: 1.972 (-1.549, 5.493) | 0.249 |
| Sulfonylureas | 1 (0.3) | 2 (0.6) | RD%: -0.282 (-1.603, 1.040) | - |
| Meglitinides | 2 (0.6) | 5 (1.4) | RD%: -0.845 (-2.559, 0.870) | 0.451 |
| TZDs | 1 (0.3) | 0 | RD%: 0.282 (-0.265, 0.828) | - |
| Alpha-glucosidase Inhibitors | 14 (3.9) | 14 (3.9) | - | - |
| DPP-4 Inhibitors | 7 (2.0) | 8 (2.3) | RD%: -0.282 (-2.423, 1.860) | 0.794 |
| SGLT2 Inhibitors | 95 (26.8) | 75 (21.1) | RD%: 5.634 (-0.507, 11.775) | 0.079 |
| GLP-1 Receptor Agonists | 3 (0.8 | 1 (0.3) | RD%: 0.563 (-0.577, 1.704) | 0.624 |
| PPAR Pan-Agonist | 1 (0.3) | 0 | RD%: 0.282 (-0.265, 0.828) | - |
| Lipid-modifying agents | 156 (43.9) | 146 (41.1) | RD%: 2.817 (-4.077, 9.711) | 0.448 |
| Statins | 148 (41.7) | 141 (39.7) | RD%: 1.972 (-4.812, 8.757) | 0.593 |
| Fibrates | 5 (1.4) | 8 (2.3) | RD%: -0.845 (-3.073, 1.382) | 0.401 |
| Cholesterol Absorption Inhibitor | 9 (2.5) | 3 (0.8) | RD%: 1.690 (-0.376, 3.755) | 0.081 |
| Immunosuppressants | 38 (10.7) | 42 (11.8) | RD%: -1.127 (-5.743, 3.489) | 0.635 |
| Glucocorticoids | 32 (9.0) | 35 (9.9) | RD%: -0.845 (-5.170, 3.479) | 0.700 |
| Hydroxychloroquine | 8 (2.3) | 4 (1.1) | RD%: 1.127 (-0.868, 3.121) | 0.244 |
| Leflunomide | 1 (0.3) | 0 | RD%: 0.282 (-0.265, 0.828) | - |
| Cyclophosphamide | 1 (0.3) | 1 (0.3) | - | - |
| Tacrolimus | 4 (1.1) | 10 (2.8) | RD%: -1.690 (-3.943, 0.562) | 0.105 |
| Mycophenolate mofetil | 6 (1.7) | 2 (0.6) | RD%: | 0.286 |
| Diuretics | 56 (15.8) | 60 (16.9) | RD%: -1.127 (-6.380, 4.127) | 0.685 |
| Loop diuretics | 32 (9.0) | 39 (11.0) | RD%: -2.000 (-6.452, 2.452) | 0.381 |
| Thiazide diuretics | 4 (1.1) | 4 (1.1) | - | - |
| Potassium-sparing diuretics | 1 (0.3) | 4 (1.1) | RD%: -0.845 (-2.447, 0.757) | 0.373 |
| ns-MRA | 21 (5.9) | 17 (4.8) | RD%: 1.127 (-2.140, 4.395) | 0.505 |
| Anemia & Bone Mineral Metabolism | 71 (20.0) | 72 (20.3) | RD%: -0.282 (-6.011%, 5.447) | 0.925 |
| Iron Supplements | 45 (12.7) | 38 (10.7) | RD%: 1.972 (-2.628, 6.573) | 0.414 |
| ESAs | 15 (4.2) | 12 (3.4) | RD%: 0.845 (-2.031, 3.721) | 0.556 |
| Roxadustat | 1 (0.3) | 2 (0.6) | RD%: -0.282 (-1.603, 1.040) | - |
| Calcitriol | 21 (5.9) | 24 (6.8) | RD%: -0.845 (-4.431, 2.741) | 0.644 |
| Calcium carbonate/ vitamin D3 | 35 (9.9) | 34 (9.6) | RD%: 0.282 (-4.018, 4.581) | 0.899 |
| Others |  |  |  |  |
| Febuxostat | 46 (13.0) | 41 (11.5) | RD%: 1.408 (-3.253, 6.069) | 0.567 |
| Anti-HBV agents | 5 (1.4) | 3 (0.8) | RD%: 0.563 (-1.103, 2.229) | 0.725 |
| Myocardial metabolic agents | 4 (1.1) | 3 (0.8) | RD%: 0.282 (-1.182, 1.745) | - |
| Antibiotics | 5 (1.4) | 4 (1.1) | RD%: 0.282 (-1.418, 1.981) | - |
| Mecobalamin | 4 (1.1) | 1 (0.3) | RD%: 0.845 (-0.577, 2.266) | 0.373 |
| Levothyroxine sodium | 37 (10.4) | 30 (8.5) | RD%: 1.972 (-2.347, 6.291) | 0.369 |
| α-Keto acid | 9 (2.5) | 15 (4.2) | RD%: -1.690 (-4.526, 1.146) | 0.213 |

BMI, body mass index; ALT, alanine aminotransferase; AST, aspartate aminotransferase; UACR, urine albumin-to-creatinine ratio; PT, prothrombin time; APTT, activated partial thromboplastin time; TT, thrombin time; INR, international normalized ratio; CVD, cardiovascular disease; CCB, calcium channel blockers; TZDs, thiazolidinediones; DPP-4, dipeptidyl peptidase-4; SGLT2, sodium-glucose cotransporter 2; GLP-1, glucagon-like peptide-1; PPAR, peroxisome proliferator-activated receptor; ns-MRA, non-steroidal mineralocorticoid receptor antagonist; ESAs, erythropoiesis-stimulating agents; SMD, standardized mean difference (Cohen’s d); Median diff, Hodges-Lehmann median difference estimate; RD, risk difference.

Continuous data are presented as mean ± SD (normally distributed) or median [IQR] (non-normally distributed). Categorical data are presented as n (%). P values for continuous variables are from t-tests or Mann-Whitney U tests, and from χ² or Fisher’s exact tests for categorical variables.

*See medical history definition.

# Table S2. Biopsy information and pathological diagnoses

| **Characteristic, n (%)** | **Vitamin C** | **Control** | **P value** |
| --- | --- | --- | --- |
|  | N=355 | N=355 |  |
| Needle insertions |  |  |  |
| 2 | 350 (98.6) | 345 (97.2) | 0.192 |
| 3 | 5 (1.4) | 10 (2.8) |  |
| Left renal biopsy | 0 | 2 (0.6) | 0.499 |
| Primary Renal Diagnosis |  |  |  |
| IgA nephropathy | 111 (31.3) | 103 (31.3) | 0.513 |
| Membranous nephropathy | 69 (19.4) | 86 (24.2) | 0.122 |
| Diabetic nephropathy | 47 (13.2) | 38 (10.7) | 0.298 |
| Hypertensive renal disease | 34 (9.6) | 21 (5.9) | 0.068 |
| Minimal change disease | 15 (4.2) | 17 (4.8) | 0.718 |
| Focal segmental glomerulosclerosis | 12 (3.4) | 15 (4.2) | 0.556 |
| Lupus nephritis | 6 (1.7) | 8 (2.3) | 0.589 |
| Other glomerulonephritides | 47 (13.2) | 51 (14.4) | 0.663 |
| Amyloidosis | 5 (1.4) | 11 (3.1) | 0.129 |
| ANCA-associated vasculitis | 6 (1.7) | 4 (1.1) | 0.524 |
| IgA vasculitis (HSP) | 6 (1.7) | 8 (2.3) | 0.589 |
| Minor glomerular abnormalities | 5 (1.4) | 3 (0.8) | 0.725 |
| Obesity-related glomerulopathy | 9 (2.5) | 8 (2.3) | 0.806 |
| Membranoproliferative glomerulonephritis | 1 (0.3) | 3 (0.8) | 0.624 |
| Proliferative glomerulonephritis with monoclonal immunoglobulin deposits | 2 (0.6) | 1 (0.3) | - |
| Monoclonal Gammopathy of Undetermined Significance | 1 (0.3) | 0 | - |
| Drug-induced kidney injury | 2 (0.6) | 3 (0.8) | - |
| C3 glomerulopathy | 0 | 2 (0.6) | 0.499 |
| C4 glomerulopathy | 0 | 1 (0.3) | - |
| Fabry nephropathy | 2 (0.6) | 0 | 0.499 |
| Light chain deposition disease | 2 (0.6) | 3 (0.8) | - |
| Thin basement membrane nephropathy | 3 (0.8) | 4 (1.1) | - |
| Thrombotic microangiopathy | 2 (0.6) | 0 | 0.499 |
| IgM nephropathy | 1 (0.3) | 0 | - |
| Other / Unclassified | 14 (3.9) | 16 (4.5) | 0.709 |
| Acute tubulointerstitial injury | 6 (1.7) | 8 (2.3) | 0.589 |
| Chronic or nonspecific glomerular/ tubulointerstitial lesions | 5 (1.4) | 5 (1.4) | - |
| Ischemic/vascular renal injury | 1 (0.3) | 3 (0.8) | 0.624 |

# Table S3. Pain VAS Scores Over Time

| **Time Point** | **Vitamin C, Median [IQR]** | **Control, Median [IQR]** | **Effect Size (95% CI)** | **P value** |
| --- | --- | --- | --- | --- |
|  | N=355 | N=355 |  |  |
| 2 hours^*^ | 0 [0, 9] | 0 [0, 6] | Median Diff: - | 0.035 |
| 12 hours^*^ | 0 [0, 7] | 0 [0, 5] | Median Diff: - | 0.064 |
| 24 hours^*^ | 0 [0, 4] | 0 [0, 3] | Median Diff:- | 0.178 |
| VAS>0 |  |  |  |  |
| 2 hours, n(%) | 161 (45.4) | 130 (36.6) | RR: 1.238 (1.036, 1.480) | 0.018 |
|  | 11 [4, 26.5] | 12 [4, 25.3] | Median Diff: -1 (-3, 2) | 0.612 |
| 12 hours | 150 (42.3) | 128 (36.1) | RR: 1.172 (0.975, 1.409) | 0.091 |
|  | 10 [5, 27] | 9 [4, 19.8] | Median Diff: 1 (1, 3) | 0.416 |
| 24 hours | 135 (38.0) | 117 (33.0) | RR: 1.154 (0.946, 1.408) | 0.158 |
|  | 6 [3, 14] | 6 [3, 13] | Median Diff: 0 (-1, 1) | 0.938 |

* Unadjusted.

# Table S4. Results of Zero-Inflated Negative Binomial Regression for Pain Intensity

| **Time Point** | **Variable** | **Count Part (Pain Intensity)** | | **P value** | **Zero-Inflated Part (Zero Probability)** | | **P value** |
| --- | --- | --- | --- | --- | --- | --- | --- |
|  |  | β (95% CI) | RR (95% CI) |  | β (95% CI) | OR (95% CI) |  |
| 2 h | Intercept | 3.152 (2.609, 3.694) | - | <0.001 | -0.705 (-1.274, -0.135) | 0.494 (0.280, 0.874) | 0.015 |
|  | Vitamin C group | 0.018 (-0.247, 0.284) | 1.018 (0.781, 1.329) | 0.893 | - | - | - |
|  | Age | -0.008 (-0.017, 0.001) | 0.992 (0.983, 1.001) | 0.072 | 0.019 (0.008, 0.030) | 1.019 (1.008, 1.031) | <0.001 |
|  | Log(θ) | -0.234 (-0.480, 0.012) | - | 0.062 | - | - | - |
| 12 h | Intercept | 3.572 (2.989, 4.155) | - | <0.001 | -0.723 (-1.302, -0.143) | 0.485 (0.272, 0.867) | 0.015 |
|  | Vitamin C group | -0.048 (-0.327, 0.230) | 0.953 (0.721, 1.259) | 0.734 | - | - | - |
|  | Age | -0.016 (-0.026, -0.006) | 0.984 (0.974, 0.994) | 0.001 | 0.021 (0.009, 0.033) | 1.021 (1.009, 1.034) | <0.001 |
|  | Log(θ) | -0.313 (-0.574, -0.052) | - | 0.019 | - | - | - |
| 24 h | Intercept | 2.809 (2.146, 3.471) | - | <0.001 | -0.461 (-1.113, 0.191) | 0.630 (0.329, 1.210) | 0.166 |
|  | Vitamin C group | -0.021 (-0.330, 0.288) | 0.979 (0.719, 1.334) | 0.894 | - | - | - |
|  | Age | -0.014 (-0.024, -0.003) | 0.986 (0.976, 0.997) | 0.013 | 0.015 (0.002, 0.028) | 1.015 (1.002, 1.028) | 0.020 |
|  | Log(θ) | -0.474 (-0.823, -0.124) | - | 0.008 | - | - | - |

RR = Rate Ratio; OR = Odds Ratio; θ = overdispersion parameter of the negative binomial distribution. Dashes (-) indicate that the variable was not included in the corresponding part of the model. The "Vitamin C group" variable is included in the count part only.

# Table S5. Adverse events during the study period (CTCAE v5.0)

| **Preferred Term, n (%)** | **Vitamin C** | **Control** |
| --- | --- | --- |
|  | N=355 | N=355 |
| Any adverse events | 15 (4.23) | 13 (3.66) |
| Gastrointestinal Disorders | 9 (2.54) | 5 (1.41) |
| Abdominal Distension | 3 (0.85) | 2 (0.56) |
| Nausea | 3 (0.85) | 2 (0.56) |
| Constipation | 1 (0.28) | 0 |
| Abdominal Pain | 0 | 1 (0.28) |
| Dyspepsia | 1 (0.28) | 0 |
| Vomiting | 1 (0.28) | 0 |
| General Conditions and Administration Site Conditions | 2 (0.56) | 2 (0.56) |
| Pyrexia | 0 | 1 (0.28) |
| Fatigue | 1 (0.28) | 1 (0.28) |
| Non-cardiac Chest Pain | 1 (0.28) | 0 |
| Respiratory, Thoracic and Mediastinal Disorders | 0 | 1 (0.28) |
| Productive Cough | 0 | 1 (0.28) |
| Musculoskeletal and Connective Tissue Disorders | 3 (0.85) | 1 (0.28) |
| Myalgia | 3 (0.85) | 1 (0.28) |
| Nervous System Disorders | 3 (0.85) | 3 (0.85) |
| Headache | 1 (0.28) | 1 (0.28) |
| Dizziness | 1 (0.28) | 0 |
| Paresthesia | 0 | 1 (0.28) |
| Stroke | 0 | 1 (0.28) |
| Presyncope | 1 (0.28) | 0 |
| Renal and Urinary Disorders | 0 | 2 (0.56) |
| Urinary Retention | 0 | 1 (0.28) |
| Pollakiuria | 0 | 1 (0.28) |
| Psychiatric Disorders | 0 | 1 (0.28) |
| Anxiety | 0 | 1 (0.28) |

AE grading followed CTCAE v5.0. Incidence (%) = (n/N) × 100, where n is the number of patients with the AE

# Table S6. Summary of Adverse Event Severity graded by CTCAE 5.0

| **Grade, n (%)** | **Vitamin C** | | **Control** | |
| --- | --- | --- | --- | --- |
|  | **Events** | **Patients** | **Events** | **Patients** |
| Any | 17 | 15 | 15 | 13 |
| 1 | 16 (94.12) | 14 (93.33) | 14 (93.33) | 12 (92.31) |
| 2 | 1 (5.88) | 1 (6.67) | 1 (6.67) | 1 (7.69) |
| 3 | 0 | 0 | 0 | 0 |
| 4 | 0 | 0 | 0 | 0 |
| 5 | 0 | 0 | 0 | 0 |

Figure S1. Association of Vitamin C treatment and participants age with postbiopsy pain intensity. Results from zero-inflated negative binomial regression models showing the association between treatment group, patient age, and Visual Analogue Scale (VAS) pain scores at 2, 12, and 24 hours postoperatively. Estimates are presented as exponentiated coefficients (exp(β)), interpreted as rate ratios (RR), with 95% confidence intervals (CI). The vertical dashed line at RR = 1.0 represents the null value (no effect). A: For the Vitamin C Group, the RR compares the treatment group to the control group on the count (pain intensity) part of the model. Values are centered around 1.0 with CIs crossing the null line, indicating no statistically significant effect of Vitamin C on pain intensity scores at any time point (all p > 0.05). B: For Age, the RR represents the multiplicative change in pain intensity per one-year increase in age, based on the count part of the model. The point estimates are consistently below 1.0 (12h & 24h: p < 0.05), indicating that older age is associated with a modest but statistically significant reduction in reported pain intensity. *p < 0.05, **p < 0.01.

2.2 Post-hoc subgroup analysis

# Table S7. Sample sizes for stratification of baseline urinary vitamin C levels

| **Stratification** | **Subgroup** | **Definition** | **N (Total=710)** |
| --- | --- | --- | --- |
| Tertiles | Low VC | Lowest tertile | 237 |
|  | Medium VC | Middle tertile | 236 |
|  | High VC | Highest tertile | 237 |
| Quartiles | Q1 (lowest) | Lowest quartile | 178 |
|  | Q2 | Second quartile | 177 |
|  | Q3 | Third quartile | 177 |
|  | Q4 (highest) | Highest quartile | 178 |

Tertiles and quartiles were defined based on the distribution of baseline urinary vitamin C concentration in the entire study cohort (N=710). Equal-sized groups were formed

# Table S8. Post-hoc subgroup analysis by baseline urinary vitamin C tertiles

| **Outcome** | **Tertile** | **Effect estimate (Intervention vs. Control)** | **95% CI** | **P value** | **Interaction P value** |
| --- | --- | --- | --- | --- | --- |
| Perirenal hematoma area,  (mm²) | Low VC | MD = 39.32 | (-25.44, 104.07) | 0.233 | 0.422 |
|  | Medium VC | MD = -50.29 | (-189.84, 89.27) | 0.477 |  |
|  | High VC | MD = 14.84 | (-60.55, 90.23) | 0.699 |  |
| VAS pain at 2h | Low VC | MD = 4.31 | (0.83, 7.80) | 0.016 | 0.185 |
|  | Medium VC | MD = -0.62 | (-5.11, 3.87) | 0.785 |  |
|  | High VC | MD = 0.78 | (-2.78, 4.34) | 0.667 |  |
| VAS pain at 12h | Low VC | MD = 5.48 | (1.09, 9.86) | 0.015 | 0.064 |
|  | Medium VC | MD = -0.50 | (-4.28, 3.27) | 0.793 |  |
|  | High VC | MD = 0.16 | (-3.36, 3.68) | 0.928 |  |
| VAS pain at 24h | Low VC | MD = 1.19 | (-1.16, 3.54) | 0.319 | 0.845 |
|  | Medium VC | MD = 0.26 | (-2.21, 2.72) | 0.838 |  |
|  | High VC | MD = 0.45 | (-1.83, 2.73) | 0.695 |  |
| Any perirenal hematoma,  (OR) | Low VC | OR = 1.59 | (0.95, 2.66) | 0.101 | 0.571 |
|  | Medium VC | OR = 1.22 | (0.73, 2.05) | 0.522 |  |
|  | High VC | OR = 1.08 | (0.65, 1.80) | 0.862 |  |

MD, mean difference (Intervention - Control); OR, odds ratio (Intervention vs. Control); CI, confidence interval.

Two-sample t-test for continuous outcomes, logistic regression for binary outcome. Interaction p-values were obtained by adding treatment × subgroup term in the model (ANCOVA for area/VAS, logistic regression for hematoma).

# Table S9. Post-hoc subgroup analysis by baseline urinary vitamin C quartiles

| **Outcome** | **Quartile** | **Effect estimate (Intervention vs. Control)** | **95% CI** | **P value** | **Interaction P value** |
| --- | --- | --- | --- | --- | --- |
| Perirenal hematoma area,  (mm²) | Q1 (lowest) | MD = 62.84 | (-4.05, 129.73) | 0.065 | 0.484 |
|  | Q2 | MD = -60.19 | (-239.54, 119.15) | 0.507 |  |
|  | Q3 | MD = -12.64 | (-82.51, 57.24) | 0.722 |  |
|  | Q4 (highest) | MD = 19.82 | (-73.89, 113.53) | 0.677 |  |
| VAS pain at 2h | Q1 | MD = 3.77 | (-0.46, 8.00) | 0.080 | 0.718 |
|  | Q2 | MD = 0.97 | (-3.98, 5.91) | 0.700 |  |
|  | Q3 | MD = 0.80 | (-3.50, 5.09) | 0.715 |  |
|  | Q4 | MD = 0.51 | (-3.91, 4.94) | 0.819 |  |
| VAS pain at 12h | Q1 | MD = 5.89 | (0.80, 10.98) | 0.024 | 0.123 |
|  | Q2 | MD = 2.38 | (-2.12, 6.88) | 0.299 |  |
|  | Q3 | MD = -1.05 | (-5.12, 3.02) | 0.610 |  |
|  | Q4 | MD = -0.42 | (-4.81, 3.96) | 0.849 |  |
| VAS pain at 24h | Q1 | MD = 1.15 | (-1.60, 3.89) | 0.411 | 0.582 |
|  | Q2 | MD = 1.79 | (-1.01, 4.59) | 0.208 |  |
|  | Q3 | MD = -0.81 | (-3.31, 1.68) | 0.520 |  |
|  | Q4 | MD = 0.41 | (-2.49, 3.32) | 0.778 |  |
| Any perirenal hematoma,  (OR) | Q1 | OR = 1.82 | (1.01, 3.32) | 0.068 | 0.364 |
|  | Q2 | OR = 1.50 | (0.83, 2.75) | 0.235 |  |
|  | Q3 | OR = 0.90 | (0.50, 1.62) | 0.830 |  |
|  | Q4 | OR = 1.14 | (0.63, 2.05) | 0.784 |  |

Abbreviations as in Supplementary Table S8. Interaction p-values were calculated using the same methods

3. Supplementary Definitions

# Medical History Definition

1. History of Hypertension: Diagnosis of hypertension by a physician at least 6 months prior to randomization, and/or current treatment with antihypertensive medication(s).

2. History of Diabetes Mellitus: Diagnosis of type 1 or type 2 diabetes mellitus by a physician at least 6 months prior to randomization, and/or current management with diet control, oral hypoglycemic agents, or insulin therapy.

3. Hepatitis B Virus (HBV) Infection:

- HBV Infection: Serologically confirmed at baseline, defined as positive Hepatitis B surface antigen (HBsAg) with or without positive HBV DNA.

- Resolved/Past HBV Infection: Defined as negative HBsAg but positive Hepatitis B core antibody (HBcAb) at bas- eline, with or without positive Hepatitis B surface antibody (HBsAb). This category excludes individuals with evidence of vaccination response only.

- Vaccination Response: Defined as negative HBsAg, negative HBcAb, but positive HBsAb.

4. History of Cardiovascular Disease (CVD):

Patients are considered to have a prior history of CVD if they meet any criterion below before randomization, as per the protocol's baseline data collection.

4.1 Coronary Artery Disease

4.1.1 Myocardial Infarction (MI): History of MI based on objective evidence (e.g., ECG changes, imaging, coronary angiography, or dynamic cardiac biomarker changes) per the ESC 2018 Fourth Universal Definition of Myocardial Infarction.

4.1.2 Unstable Angina or Angina Requiring Hospitalization: Documented by hospital records with supporting ECG changes and/or coronary angiography evidence.

4.1.3 Coronary Revascularization: Documented history of procedure (e.g., PCI or CABG).

4.2 Heart Failure

Clinical diagnosis per ESC or ACC/AHA guidelines, with symptoms/signs (e.g., dyspnea, edema) and objective imaging evidence (e.g., echocardiography showing LVEF < 40%).

4.3 Cerebrovascular Disease

- Stroke: History of acute cerebral infarction or hemorrhage confirmed by imaging (CT/MRI).

- Transient Ischemic Attack (TIA): History of TIA diagnosed by a neurologist based on typical symptoms.

4.4 Peripheral Artery Disease

Clinical symptoms (e.g., claudication) with objective evidence (e.g., ABI < 0.9, or vascular imaging/revascularization records).

4.5 Significant Valvular Heart Disease

Echocardiographically confirmed moderate or greater valvular stenosis/regurgitation (e.g., severe aortic stenosis) deemed clinically significant.

# Biopsy Procedure

All biopsy procedures were performed by a single experienced senior specialist and her dedicated professional team to ensure procedural standardization and minimize inter-operator variability.

Under ultrasound guidance, a renal biopsy of the lower pole was performed with the patient in the lateral decubitus position. After routine skin disinfection and draping, layered local anesthesia was administered using 5 mL of 2% lidocaine up to the renal fat capsule. Renal tissue samples were acquired with a 16G Tru‑Cut needle and submitted for light microscopy, electron microscopy, and immunofluorescence examination.

After the biopsy, the patient remained in the procedure room for manual compression at the puncture site for approximately 10 minutes. Color Doppler flow imaging (CDFI) or contrast-enhanced ultrasound (CEUS) was then performed to confirm the absence of significant active bleeding in the perirenal space or the collecting system. Only after confirmation was the patient allowed to leave the procedure room. The puncture site was disinfected and covered with gauze. A compression pad was placed over the biopsy side of the lower back with the assistance of nursing staff, and the abdominal binder was secured to keep the pad in place. The patient was then placed on strict supine bed rest for 6 to 8 hours.
