# Supplementary material for: Effect of Vitamin C on bleeding and pain after kidney biopsy: a randomized controlled trial
Source: BMC Med. 2026 Jul 3;24:515. doi: 10.1186/s12916-026-05037-x (PMC13613656; doi:10.1186/s12916-026-05037-x)
Supplement: Supplementary file 2 [file 12916_2026_5037_MOESM2_ESM.docx]

Randomized Controlled Study on Vitamin C for Reducing Complications after Renal Biopsy

Leading Institution: Xijing Hospital

Principal Investigator: He Lijie

Responsible Department: Department of Nephrology, Xijing Hospital

Contact Number: 029-89661611

Study Duration: June 01, 2025 - December 31, 2026

Version Number: V2.0

Version Date: June 16, 2025

**目录**

[Administrative Information	1](#_Toc4711)

[Synopsis	2](#_Toc17377)

[1. Study Background	4](#_Toc27505)

[2. Study Background	5](#_Toc17851)

[3. Study Background	6](#_Toc29980)

[4. Patient Selection	6](#_Toc22696)

[(I) Inclusion Criteria	6](#_Toc5031)

[(II) Exclusion Criteria	6](#_Toc16818)

[(III) Criteria for Study Termination	8](#_Toc10927)

[5. Research Methods and Technical Route	8](#_Toc9679)

[(I) Methods and Interventions	8](#_Toc9172)

[(II) Sample Size	9](#_Toc26243)

[(III) Randomization	9](#_Toc1901)

[(IV) Data Collection and Follow-up	10](#_Toc19518)

[(V) Technical Route	13](#_Toc8144)

[6. Outcomes and Measurement Time Points	13](#_Toc13563)

[(I) Primary Endpoints	13](#_Toc9219)

[(II) Secondary Endpoints	14](#_Toc28860)

[7. Potential adverse reactions	15](#_Toc3283)

[8. Quality Control and Quality Assurance	16](#_Toc703)

[9. Statistical Analysis	16](#_Toc3988)

[10. Ethics	17](#_Toc30132)

[11. Study Timeline	17](#_Toc21548)

[12. Study Personnel	18](#_Toc8753)

[13. References	18](#_Toc26633)

**Administrative Information**

**1.Trial Registration**

This trial will be registered at the Chinese Clinical Trial Registry.

**2.Study Sponsor**

The sponsor of this study is Professor He Lijie's research group at Xijing Hospital, affiliated with Air Force Medical University.

**3.Study Protocol Development**

This study protocol was developed by the study initiator, Professor He Lijie's research group.

**4.Participating Teams and Responsibilities**

| **Team** | **Institution** | **Role and Responsibilities** |
| --- | --- | --- |
| Professor He Lijie's Team | The First Affiliated Hospital of Air Force Medical University | Coordinating overall study implementation, study protocol design, daily research operations, data collection, convening investigator meetings, report writing, and making decisions regarding submission and publication of study results. |

# **Synopsis**

| **Item** | **Content** |
| --- | --- |
| Title | Randomized Controlled Study on Vitamin C for Reducing Complications after Renal Biopsy |
| Objective | To evaluate the impact of intravenous vitamin C intervention administered peri-biopsy on the incidence of post-biopsy bleeding and pain complications. This trial aims to explore a novel approach for improving perirenal hematoma, bleeding risk, and local pain in patients after renal biopsy. The goal is to provide new ideas and solutions for reducing biopsy-related complications, safeguarding patient health, improving postoperative comfort, and optimizing the treatment and management of renal biopsy patients. |
| Design | Prospective, open-label, randomized controlled clinical trial |
| Sample Size | 710 |
| Patient Selection | **Inclusion Criteria:**   1. Written informed consent obtained; 2. Age 18-80 years; 3. Undergoing percutaneous ultrasound-guided native renal biopsy.   **Exclusion Criteria:**   1. Allergy or hypersensitivity to components of the vitamin injection preparation; 2. Significant bleeding tendency; 3. Severe uncontrolled hypertension; 4. Atrophic kidney (length <8cm), solitary kidney, polycystic kidney, wandering kidney, or kidney positioned too high; 5. Severe comorbidities or poor systemic condition; 6. Poor positioning; 7. Pregnant or lactating women; 8. Oliguric patients (urine output <400ml/24h); 9. Thalassemia, cystinuria, glucose-6-phosphate dehydrogenase deficiency, and other diseases affecting coagulation function. |
| Treatment Protocol | **Control Group:**Receives only conventional intervention.  **Intervention Group:**In addition to conventional intervention, receives 20mg/kg Vitamin C Injection + 100ml 0.9% Sodium Chloride Injection. |
| Study Endpoints | **Primary Endpoints:** perirenal hematoma area and local pain VAS score.  **Secondary Endpoints:** Incidence of all bleeding events, pain, and adverse events (AE). |
| Statistical Methods | Continuous variables conforming to a normal distribution are presented as mean ± standard deviation, with between-group means compared using t-tests. Those not conforming to normal distribution are presented as median (interquartile range) [M(P25, P75)] and analyzed using non-parametric tests. Categorical variables are expressed as percentages, with between-group rates compared using χ² tests. |
| Duration | June 2025 to December 2026 |

**1. Study Background**

Percutaneous Renal Biopsy (PRB) is a crucial technical procedure for diagnosing kidney diseases. By obtaining renal tissue for pathological examination, it provides key evidence for clarifying etiology, guiding treatment, and assessing prognosis. However, as an invasive procedure, the incidence of post-PRB complications remains non-negligible, with perirenal hematoma (incidence approximately 3-85%), gross hematuria (3.5%), and local pain (4.3%) being the most common [1]. Although advancements in imaging guidance have significantly reduced the incidence of severe complications (such as death, major bleeding requiring interventional therapy or transfusion), minor complications can still prolong hospitalization, increase medical costs, and affect patients' postoperative quality of life.

Current preventive strategies for post-PRB complications primarily focus on optimizing procedural techniques (e.g., biopsy needle selection, puncture path planning) and postoperative management (e.g., bed rest, application of hemostatic drugs). However, the efficacy of hemostatic agents like tranexamic acid and desmopressin remains controversial, and there is a lack of reliable evidence regarding the safety and hemostatic effectiveness of desmopressin in advanced kidney disease [2]. Therefore, exploring safe, effective, and economical novel interventions to further reduce the risk of post-PRB complications is an important direction for clinical research.

Vitamin C (ascorbic acid), as a natural antioxidant, has garnered attention for its role in tissue repair and vascular protection. Its potential mechanisms may involve: ① Reducing oxidative stress by scavenging free radicals, protecting endothelial cell function, and decreasing vascular permeability; ② Promoting collagen synthesis and enhancing the stability of the vascular basement membrane; ③ Inhibiting the release of inflammatory factors and alleviating local tissue inflammation [3-5]. Regarding bleeding reduction, previous clinical studies have shown that perioperative intravenous vitamin C supplementation can reduce blood loss during total abdominal hysterectomy [6] and post-polypectomy bleeding [7]. Another meta-analysis indicated that among different interventions to reduce transfusion requirements during minimally invasive myomectomy, vitamin C was second only to oxytocin in effectiveness, though not significantly [8]. Notably, study results regarding the impact of vitamin C/ascorbic acid on bleeding volume and incidence of bleeding events are heterogeneous [9], which may be related to surgery type and dosage differences. Regarding pain alleviation, multiple studies suggest vitamin C can significantly reduce postoperative pain scores. One randomized controlled trial found patients receiving intravenous vitamin C had significantly lower pain scores within 24 hours postoperatively compared to control and N-acetylcysteine groups [10]. Another meta-analysis also showed significantly lower pain scores at 1-2 hours and 24 hours postoperatively in the vitamin C group compared to control [9]. However, there is currently no systematic report on the application of vitamin C after minimally invasive procedures like PRB. Therefore, further large-scale studies are necessary to further validate the safety and efficacy of vitamin C in reducing postoperative complications such as bleeding and local pain.

Based on the above background, this study aims to investigate the efficacy and safety of perioperative intravenous vitamin C supplementation on post-PRB bleeding and pain complications through a randomized controlled trial. If proven effective, this intervention will provide new insights for optimizing perioperative management in renal biopsy patients and offer evidence-based support for expanding the clinical application of vitamin C, holding significant clinical and social value.

**2. Study Background**

To evaluate the impact of intravenous vitamin C intervention administered peri-biopsy on the incidence of post-biopsy bleeding and pain complications, with postoperative perirenal hematoma area and local pain as primary outcome measures, and bleeding events and adverse events (AE) as secondary outcome measures.

This trial aims to explore a novel method for improving perirenal hematoma, bleeding risk, and local pain in patients after renal biopsy. The goal is to provide new ideas and solutions for reducing biopsy-related complications, safeguarding patient health, improving postoperative comfort, and optimizing the treatment and management of renal biopsy patients.

**3. Study Background**

This is a prospective, open-label, randomized controlled clinical trial initiated by the team of Associate Professor He Lijie from the Department of Nephrology, Xijing Hospital. Eligible patients will be randomly allocated in a 1:1 ratio to the intervention group and the control group.

The study plans to recruit 710 patients requiring renal biopsy and willing to accept the perioperative vitamin C intervention protocol at Xijing Hospital, Air Force Medical University. The intervention group will receive vitamin C, while the control group will receive no additional intervention, with 355 subjects recruited for each group. The study will be conducted from June 2025 to December 2026.

**4. Patient Selection**

**(I) Inclusion Criteria**

(1) Written informed consent obtained.
(2) Age 18-80 years.
(3) Scheduled to undergo percutaneous ultrasound-guided native renal biopsy.

**(II) Exclusion Criteria**

(1) Allergy or hypersensitivity to components of the vitamin injection preparation.
(2) Significant bleeding tendency, meeting any of the following conditions:
a. Recent use of anticoagulant or antiplatelet agents,

- Anticoagulants: Warfarin discontinued <5 days, unfractionated heparin (UFH) <6 hours, low molecular weight heparin (LMWH) <1 day, direct oral anticoagulants (DOACs) <5 days.

- Antiplatelet agents: Aspirin discontinued <7 days, clopidogrel/prasugrel <5 days, ticagrelor <3 days, dipyridamole <5 days, or indobufen <1 day [11, 12].

b. Markedly abnormal coagulation parameters,

- PT < 4.7s or > 13.5s,

- APTT < 16.6s or > 53.6s,

- TT < 11.5s or > 22.2s.

(3) Severe uncontrolled hypertension, with pre-operative systolic blood pressure > 180mmHg despite antihypertensive medication.
(4) Atrophic kidney (length <8cm), solitary kidney, polycystic kidney, wandering kidney (kidney position cannot be fixed during biopsy), or kidney positioned too high.
(5) Severe comorbidities or poor systemic condition, including: renal tumor, severe cardiac insufficiency (NYHA class III–IV), severe thrombocytopenia (<80×10⁹/L), or severe anemia (<60g/L).
(6) Poor positioning: Patients unable to maintain prone position due to massive pleural/ascitic effusion, morbid obesity, or respiratory diseases.
(7) Pregnant or lactating women.
(8) Oliguric patients (urine output <400ml/24h).
(9) Thalassemia, cystinuria, glucose-6-phosphate dehydrogenase deficiency, and other diseases affecting coagulation function.

**(III) Criteria for Study Termination**

Criteria for participant withdrawal include:

(1) Occurrence of a serious AE related to the study drug or renal biopsy, including severe allergic reactions, significant changes in blood pressure/heart rate, or thromboembolic events.
(2) Participant lost to follow-up or death.
(3) Participant or their legal guardian voluntarily requests withdrawal.

The withdrawal rate will be calculated at the end of the study. Reasons and dates for withdrawal will be recorded in detail. For participants who withdraw, appropriate treatment will be provided based on the judgment of the research staff at the participating center, and follow-up for treatment outcomes will continue.

**5. Research Methods and Technical Route**

**(I) Methods and Interventions**

All eligible patients will be randomly assigned to the intervention group (20mg/kg Vitamin C + 100mL normal saline) or the control group (no additional intervention). All patients will routinely receive conventional therapeutic interventions, including prophylactic hemostatic drugs. Both groups will receive two perioperative administrations, 24 hours apart, via intravenous injection at 6 hours pre-biopsy and 18 hours post-biopsy (Figure 1).

**Figure 1. Study Flowchart**

All patients will undergo renal ultrasound 24 hours post-biopsy to assess perirenal hematoma size. All patients will complete self-assessment using the Visual Analogue Scale (VAS) for pain at 2, 12, and 24 hours post-biopsy.

**(II) Sample Size**

Based on the results of the research group's previous multi-center RCT "Ascorbic Acid (Vitamin C) for Reducing Bleeding Complications after Renal Biopsy", among 310 subjects, 199 (64.2%) had renal hematoma detected on ultrasound 24 hours postoperatively. The hematoma area was 235.75 ± 157.90 mm² in the Vitamin C group and 289.82 ± 244.15 mm^2^ in the control group. Setting α=0.05, β=0.1, two-sided test, with 1:1 allocation, calculation using PASS (V15.0.5) indicated a minimum requirement of 616 patients (308 per group) without considering sample loss.

Sensitivity analysis scenario: Considering the effect size might be 20% smaller than expected (i.e., reduction in effect size proportion), while maintaining 80% target power, using the sample size formula for two independent samples t-test (large sample z approximation), the formula is:

$$n_{each\_group}=\frac{2\left( z_{1-\alpha/2}+z_{1-\beta} \right)^{2}}{d^{2}}$$

Where: z_{1-α/2} = z_{0.975} = 1.96 (two-sided test, α=0.05), z_{1-β} = z_{0.80} = 0.8416 (power 80%), d is Cohen's d (standardized effect size). Total sample size = 2 × n_each_group.

Sensitivity adjustment: The reduction proportion refers to the reduction in Cohen's d (i.e., new effect size d_new = d × (1 - reduction proportion)). Therefore, anticipating a 20% reduction in effect size, calculating and rounding up results in 355 patients per group, requiring a total sample size of 710.

**(III) Randomization**

This study will use a centralized random sequence provided by a statistician to allocate subjects. The statistician will use SPSS (V27.0) to generate a random number table. According to this table, corresponding group codes will be numbered.

Subjects will receive a sequential number based on their time of confirmed entry into the study, which corresponds to the group code number.

**(IV) Data Collection and Follow-up**

(1) Data Collection. All subjects need to complete data collection for informed consent, medical history, physical examination, ECG/echocardiography assessment, routine tests, concomitant medication records, endpoint outcome assessments, etc., at different study stages (Table 1).

**Table 1. Study Activities and Assessment Schedule**

| **Plan** | **Screening** | **Baseline** | **Follow-up 1** | **Follow-up 2** | **Follow-up 3** | **EOS**  **Follow-up** | **PT**  **Follow-up** |
| --- | --- | --- | --- | --- | --- | --- | --- |
| Time | ≤2 weeks | Day -2 | Hour 2 | Hour 12 | Hour 24 | Day 2 | 4 weeks |
| Informed Consent | × |  |  |  |  |  |  |
| Inclusion/Exclusion Criteria | × |  |  |  |  |  |  |
| Demographics | × |  |  |  |  |  |  |
| Past Medical History | × |  |  |  |  |  |  |
| Medication History | × |  |  |  |  |  |  |
| Concomitant Medications | × | × | × | × | × | × | × |
| Randomization |  | × |  |  |  |  |  |
| Drug Management Responsibility |  | × |  |  |  |  |  |
| Study Drug Allocation |  | × |  |  |  |  |  |
| Study Drug Administration |  | × |  |  |  |  |  |
| Physical Exam | × | × |  |  |  |  |  |
| Weight, Height | × |  |  |  |  |  |  |
| Vital Signs | × | × |  |  |  | × |  |
| ECG/Echocardiography | × |  |  |  |  |  |  |
| Renal Ultrasound |  |  |  |  | × |  |  |
| Blood Sample | × | × |  |  |  |  | × |
| Urine Sample | × | × |  |  |  | × | × |
| Endpoint Assessment |  |  | × | × | × | × |  |
| AE Assessment |  |  | × | × | × | × | × |

a. Screening Stage. Screen potential subjects based on inclusion/exclusion criteria. Eligible subjects (or their legal representatives) sign informed consent. Collect demographic information, past medical history, medication history, concomitant medications, physical examination, vital signs, etc. Complete pre-operative 12-lead ECG, echocardiography, renal ultrasound. Collect venous blood samples for complete blood count, liver/kidney function, lipids, blood glucose, electrolytes. Collect clean-catch morning midstream urine for urinalysis and urine sediment. Collect 24-hour urine for urine protein quantification.
b. Baseline. Randomize enrolled subjects. Record concomitant medications. Record physical examination and vital signs data. Collect venous blood samples for coagulation function, inflammatory markers (C-reactive protein, procalcitonin, interleukin-6). Collect a single discontinuous urine sample for three-glass urine test. Collect clean-catch morning midstream urine for urinary microalbumin/creatinine ratio and blood/urine vitamin C detection.
c. Follow-up 1 and 2. Record concomitant medications. Guide subjects to complete VAS self-assessment. Assess AEs.
d. Follow-up 3. Complete postoperative renal ultrasound and urine vitamin C detection. Record concomitant medications. Guide subjects to complete VAS self-assessment. Assess AEs.
e. End of Study (EoS) Follow-up. Record concomitant medications and vital signs data. Assess AEs (secondary outcome).
f. Post-Treatment (PT) Follow-up. Record concomitant medications. Assess AEs (secondary outcome). Collect venous blood samples for complete blood count, liver/kidney function, lipids, electrolytes; add blood glucose test for diabetic patients. Collect clean-catch morning midstream urine for urinalysis and urine sediment. Collect 24-hour urine for urine protein quantification.
(2) Demographic Data. Includes name, gender, date of birth, ethnicity, education level, occupation, marital status, current address, contact information.
(3) Laboratory Tests. Complete Blood Count: White blood cells, neutrophils, lymphocytes, monocytes, eosinophils, basophils, red blood cell count, hemoglobin, platelets. Liver/Kidney Function: ALT, AST, albumin, urea, creatinine, uric acid, cystatin C, eGFR (CKD-EPI). Lipids: Cholesterol, triglycerides, HDL, LDL. Coagulation Function: PT, APTT, Fib, TT, D-Dimer, INR.

If multiple laboratory results exist, the one closest to the renal biopsy time will be selected.

(4) Biopsy Procedure. The patient will undergo local anesthesia-guided biopsy of the lower pole of the right kidney in the lateral decubitus position under ultrasound localization. After routine skin disinfection and draping, 5mL of 2% lidocaine is administered for local anesthesia layer by layer to the renal fat capsule. A 16G TRU-CUT needle is inserted perpendicularly to obtain satisfactory renal tissue, which is then sent for light microscopy, electron microscopy, and immunofluorescence. The puncture wound is disinfected, covered with gauze, and pressure bandaged with an abdominal binder. The patient is then returned to the ward by wheelchair and kept supine for 8 hours.
(5) Concomitant Medication Record. Usage of antihypertensive, lipid-lowering, hypoglycemic, immunomodulatory, adjuvant therapy, and other relevant medications.
(6) Strategies to Ensure Data Collection Integrity
4. Routine Clinical Visit Data Collection: This study will use paper-based Case Report Forms (CRFs) for data collection, establish an electronic database for data management, employ double data entry with comparison for data entry and verification, and conduct regular data verification and cleaning. During the study, research personnel will, in principle, review trial data monthly to ensure internal consistency.
5. Sending Reminders to Subjects: Automated reminder messages will be sent to subjects in advance via WeChat group, SMS, or phone calls to minimize sample loss due to loss to follow-up and/or withdrawal.

**(V) Technical Route**

**Figure2. Technical Route**

**6. Outcomes and Measurement Time Points**

(I) Primary Endpoints

1. perirenal Hematoma Outcome. The area of perirenal hematoma on ultrasound imaging 24 hours post-biopsy in both groups. The estimation method for perirenal hematoma area is the product of the two diameters of the hypoechoic area around the kidney on two-dimensional ultrasound images.

2. Local Pain Outcome. Pain VAS score levels at 2, 12, and 24 hours postoperatively in both groups.

- VAS Score Details

a. Tool Description: The pain VAS used in the study is a continuous scale consisting of a 10 cm (100 mm) horizontal line with verbal descriptors at both ends: "No Pain" and "Worst Pain Imaginable". To avoid central tendency bias towards specific numbers, no numeric or verbal markings are placed along the intermediate scale.

b. Assessment Time: Patients are required to self-assess the current pain level at the puncture site at 2, 12, and 24 hours postoperatively.

c. Implementation Method: The pain VAS is completed by the patient independently. Subjects need to make a mark perpendicular to the VAS line at the position representing their pain intensity.

d. Scoring Method: The researcher uses a ruler to measure the distance in millimeters from the "No Pain" endpoint to the patient's mark, resulting in a score range of 0-100.

e. Result Interpretation: A higher score indicates greater pain intensity. Based on postoperative pain level, it can be categorized into four grades: No pain (0-4 mm), Mild (5-44 mm), Moderate (45-74 mm), and Severe (75-100 mm) [13].

(II) Secondary Endpoints

(1) All Bleeding Event Outcomes，including:

a. Incidence of perirenal hematoma, including overall incidence and incidence of mild (≤200mm²), moderate (>200 and ≤400mm²), and severe (>400mm²) perirenal hematoma;
b. Gross hematuria not requiring intervention;
c. Bleeding events requiring transfusion (red blood cells) intervention;
d. Bleeding events requiring surgical or interventional procedure intervention.
(2) Local Pain Incidence. Incidence of mild, moderate, and severe pain at 2, 12, and 24 hours postoperatively.
(3) Adverse Event (AE) Outcomes.

Incidence of AEs during the study period. Recording period: from first Vitamin C injection (-6 hours) to EoS follow-up (48 hours post-biopsy).

AE severity will be graded according to the Common Terminology Criteria for Adverse Events (CTCAE) Version 5.0:

- Grade 1: Mild; asymptomatic or mild symptoms; clinical or diagnostic observations only; intervention not indicated.

- Grade 2: Moderate; minimal, local or noninvasive intervention indicated; limiting age-appropriate instrumental Activities of Daily Living (ADL).

- Grade 3: Severe or medically significant but not immediately life-threatening; hospitalization or prolongation of hospitalization indicated; disabling; limiting self-care ADL.

- Grade 4: Life-threatening consequences; urgent intervention indicated.

- Grade 5: Death related to AE.

**7. Potential adverse reactions**

Potential adverse reactions associated with vitamin C use:

(1) Allergy to drug components or excipients, clinical symptoms include rash, pruritus, chills, high fever, chest tightness, dyspnea, cyanosis, cold extremities, skin mottling, etc.

(2) Rapid intravenous administration may cause dizziness, nausea, drowsiness, headache.

(3) Overdose of Vitamin C Injection (1-4g/day) may cause diarrhea, rash, increased gastric acid, gastroesophageal reflux, and sometimes urinary stones, deep vein thrombosis, intravascular hemolysis or coagulation, and may reduce leukocyte phagocytic ability. Daily doses exceeding 5g may lead to hemolysis, which can be fatal in severe cases. High doses during pregnancy may cause infantile scurvy.

(4) Long-term high-dose use may occasionally cause urate, cystine, or oxalate stones.

(5) Long-term daily use of 2-3g may cause scurvy upon withdrawal.

(6) Long-term high-dose ingestion may also cause diarrhea, flatulence, increased excretion of iron, calcium, and manganese in urine, and increased iron absorption leading to iron overload, etc. [14].

**8. Quality Control and Quality Assurance**

Investigators will be responsible for screening subjects according to inclusion and exclusion criteria, recording study data in Case Report Forms (CRFs), and entering data into the electronic database. They must also closely monitor contact with participants according to the schedule to avoid enrollment of ineligible participants, voluntary withdrawals, participants not receiving treatment, or cross-over treatment between groups.

Given the high safety profile of the intervention drug Vitamin C and the short study duration, a Data and Safety Monitoring Board (DSMB) will not be established. This study has an independent statistician responsible for research monitoring, randomization, data management, and statistical analysis.

After research quality control procedures are completed and confirmed by the PI, the database will be locked.

**9. Statistical Analysis**

All statistical analyses will be performed based on the Intention-To-Treat (ITT) principle, regardless of poor compliance, mid-study withdrawal, or loss to follow-up. We will conduct independent observational analyses of the two treatment protocols and report the trial results truthfully. Statistical analysis will be performed using SPSS v27.0 or R software.

(1) Continuous Variables.

Normality will be tested using the Shapiro-Wilk test and visual inspection of QQ plots, and homogeneity of variance using Levene's test . For data conforming to normal distribution, results will be reported as mean ± standard deviation (Mean ± SD), with between-group comparisons using independent samples t-test. For data not conforming to normal distribution, results will be reported as median [interquartile range] (median, [IQR]), with between-group comparisons using Mann-Whitney U test.

(2) Categorical Variables

Reported as frequency (percentage). Between-group comparisons will use χ² test; Fisher's exact test will be used when any expected frequency is <5.

(3) Primary Endpoint Analysis

a. perirenal Hematoma Area (24h post-biopsy): This single-time-point continuous outcome will be compared between groups using the methods defined in Section 9 (1).

Sensitivity Analysis (if necessary): An Analysis of Covariance (ANCOVA) may be performed, adjusting for relevant baseline covariates.

b. Local Pain VAS Score (2, 12, 24h post-biopsy): Scores at each time point will be compared independently between groups. P-values will be adjusted for multiple comparisons using the Bonferroni method.

Sensitivity Analysis (if necessary): If a high prevalence of zero pain scores exists, a zero-inflated negative binomial model will be considered to adjust for baseline confounders.

(4) Secondary Endpoint Analysis

The distribution of perinephric hematoma severity (mild/moderate/severe) will be compared using the Cochran-Mantel-Haenszel test for trend to assess for a group difference in the ordinal trend.

Binary outcomes (e.g., incidence of gross hematuria, intervention-requiring bleeding) will be compared using the χ² or Fisher's exact test.

Overall Adverse Event (AE) incidence will be compared using the χ² test. Comparisons of specific AE types are considered exploratory.

**10. Ethics**

The clinical research will comply with the World Medical Association's Declaration of Helsinki and other relevant regulations. Before study initiation, the trial protocol must be approved by the Ethics Committee. Prior to enrollment, the investigator is responsible for fully and comprehensively explaining the study's purpose, procedures, potential benefits, and risks to the subject or their legal representative, and obtaining written informed consent. Subjects should understand they have the right to withdraw from the study at any time. Informed consent forms will be retained as clinical research documents for reference. The study will protect participants' personal privacy and data confidentiality.

**11. Study Timeline**

- June 01, 2025 - August 31, 2025: Preliminary preparation, study protocol design, investigator meetings, Ethics Committee review, trial registration.

- September 01, 2025 - September 30, 2026: Trial initiation, patient enrollment and follow-up, monitor visits, data collection, management of blood samples and drug AEs.

- October 01, 2026 - December 31, 2026: Data consolidation, document archiving, statistical analysis, manuscript writing and publication, reporting of results.

**12. Study Personnel**

| **Name** | **Title/Specialty** | **Tasks** | **GCP Training (Yes/No)** |
| --- | --- | --- | --- |
| He Lijie | Associate Professor / Clinical | Study design, patient diagnosis/treatment, quality control | Yes |
| Li Jipeng | Graduate Student / Clinical | Screening, enrollment, randomization | Yes |
| Zha Yang | Physician / Clinical | Treatment, AE assessment | Yes |
| Jia Qing | Physician / Clinical | Treatment, AE assessment | Yes |
| Chen Ye | Resident Physician | Patient follow-up | Yes |
| Xu Xiaomeng | Graduate Student | Patient follow-up | Yes |
| Wu Mengting | Graduate Student | Patient follow-up | Yes |
| Zhao Fang | Graduate Student | Biospecimen collection, testing | Yes |
| Lu Rui | Resident Physician | Treatment, AE assessment | Yes |

**13. References**

[1] POGGIO E D, MCCLELLAND R L, BLANK K N, et al. Systematic Review and Meta-Analysis of Native Kidney Biopsy Complications [J]. Clin J Am Soc Nephrol, 2020, 15(11): 1595-602.

[2] WANG C, LEBEDEVA V, YANG J, et al. Desmopressin to reduce periprocedural bleeding and transfusion: a systematic review and meta-analysis [J]. Perioper Med (Lond), 2024, 13(1): 5.

[3] SPOELSTRA-DE MAN A M E, ELBERS P W G, OUDEMANS-VAN STRAATEN H M. Making sense of early high-dose intravenous vitamin C in ischemia/reperfusion injury [J]. Crit Care, 2018, 22(1): 70.

[4] MAY J M, HARRISON F E. Role of vitamin C in the function of the vascular endothelium [J]. Antioxidants & redox signaling, 2013, 19(17): 2068-83.

[5] DU Y T, LONG Y, TANG W, et al. Prooxidative inhibition against NF-kappaB-mediated inflammation by pharmacological vitamin C [J]. Free Radic Biol Med, 2022, 180: 85-94.

[6] FARAHANI-JAM S, YAGHMAEI M, VAHDAT SHARIATPANAHI Z. Effect of intravenous ascorbic acid administration on hemorrhage and wound complications in total abdominal hysterectomy: A prospective randomized clinical trial [J]. Clin Nutr ESPEN, 2022, 49: 74-8.

[7] VAHDAT SHARIATPANAHI Z, SHAHBAZI S, SHAHBAZI E. Ascorbic Acid to Prevent Postpolypectomy Bleeding in the Colon: A Randomized Controlled Trial [J]. Asian J Endosc Surg, 2022, 15(1): 103-9.

[8] SAMY A, RASLAN A N, TALAAT B, et al. Perioperative nonhormonal pharmacological interventions for bleeding reduction during open and minimally invasive myomectomy: a systematic review and network meta-analysis [J]. Fertil Steril, 2020, 113(1): 224-33 e6.

[9] HUNG K C, LIN Y T, CHEN K H, et al. The Effect of Perioperative Vitamin C on Postoperative Analgesic Consumption: A Meta-Analysis of Randomized Controlled Trials [J]. Nutrients, 2020, 12(10).

[10] RACHANA N D, RANGANATH N, PALLAVI V R, et al. Effect of Intravenous Vitamin C and N Acetylcysteine on Postoperative Pain and Opioid Consumption after Laparoscopic Gynaecologic Oncosurgeries: A Randomised Controlled Study [J]. Journal of Clinical and Diagnostic Research, 2024.

[11] HOSPITAL PHARMACY PROFESSIONAL COMMITTEE OF THE CHINESE PHARMACEUTICAL A. [Chinese expert consensus on home management of oral anticoagulants(2024 edition)] [J]. Zhonghua yi xue za zhi, 2024, 104(28): 2595-612.

[12] TOMASELLI G F, MAHAFFEY K W, CUKER A, et al. 2020 ACC Expert Consensus Decision Pathway on Management of Bleeding in Patients on Oral Anticoagulants: A Report of the American College of Cardiology Solution Set Oversight Committee [J]. J Am Coll Cardiol, 2020, 76(5): 594-622.

[13] HAWKER G A, MIAN S, KENDZERSKA T, et al. Measures of adult pain: Visual Analog Scale for Pain (VAS Pain), Numeric Rating Scale for Pain (NRS Pain), McGill Pain Questionnaire (MPQ), Short-Form McGill Pain Questionnaire (SF-MPQ), Chronic Pain Grade Scale (CPGS), Short Form-36 Bodily Pain Scale (SF-36 BPS), and Measure of Intermittent and Constant Osteoarthritis Pain (ICOAP) [J]. Arthritis Care Res (Hoboken), 2011, 63 Suppl 11: S240-52.

[14] LYKKESFELDT J, CARR A C, TVEDEN-NYBORG P. The pharmacology of vitamin C [J]. Pharmacol Rev, 2025, 77(2): 100043
